# Fob1 and Fob2 Proteins Are Virulence Determinants of *Rhizopus oryzae* via Facilitating Iron Uptake from Ferrioxamine

**DOI:** 10.1371/journal.ppat.1004842

**Published:** 2015-05-14

**Authors:** Mingfu Liu, Lin Lin, Teclegiorgis Gebremariam, Guanpingsheng Luo, Christopher D. Skory, Samuel W. French, Tsui-Fen Chou, John E. Edwards, Ashraf S. Ibrahim

**Affiliations:** 1 Division of Infectious Diseases, Los Angeles Biomedical Research Institute at Harbor-University of California, Los Angeles (UCLA) Medical Center, Torrance, California, United States of America; 2 David Geffen School of Medicine at University of California, Los Angeles, Los Angeles, California, United States of America; 3 National Center for Agricultural Utilization Research, United States Department of Agriculture (USDA), Peoria, Illinois, United States of America; 4 Department of Pathology, David Geffen School of Medicine at University of California, Los Angeles, Los Angeles, California, United States of America; 5 Division of Medical Genetics, Department of Pediatrics, Harbor-University of California, Los Angeles Medical Center and Los Angeles Biomedical Research Institute, Torrance, California, United States of America; Geisel School of Medicine at Dartmouth, UNITED STATES

## Abstract

Dialysis patients with chronic renal failure receiving deferoxamine for treating iron overload are uniquely predisposed for mucormycosis, which is most often caused by *Rhizopus oryzae*. Although the deferoxamine siderophore is not secreted by *Mucorales*, previous studies established that *Rhizopus* species utilize iron from ferrioxamine (iron-rich form of deferoxamine). Here we determined that the CBS domain proteins of Fob1 and Fob2 act as receptors on the cell surface of *R*. *oryzae* during iron uptake from ferrioxamine. Fob1 and Fob2 cell surface expression was induced in the presence of ferrioxamine and bound radiolabeled ferrioxamine. A *R*. *oryzae* strain with targeted reduced Fob1/Fob2 expression was impaired for iron uptake, germinating, and growing on medium with ferrioxamine as the sole source of iron. This strain also exhibited reduced virulence in a deferoxamine-treated, but not the diabetic ketoacidotic (DKA), mouse model of mucormycosis. The mechanism by which *R*. *oryzae* obtains iron from ferrioxamine involves the reductase/permease uptake system since the growth on ferrioxamine supplemented medium is associated with elevated reductase activity and the use of the ferrous chelator bathophenanthroline disulfonate abrogates iron uptake and growth on medium supplemented with ferrioxamine as a sole source of iron. Finally, *R*. *oryzae* mutants with reduced copies of the high affinity iron permease (*FTR1*) or with decreased *FTR1* expression had an impaired iron uptake from ferrioxamine *in vitro* and reduced virulence in the deferoxamine-treated mouse model of mucormycosis. These two receptors appear to be conserved in *Mucorales*, and can be the subject of future novel therapy to maintain the use of deferoxamine for treating iron-overload.

## Author Summary

Deferoxamine is an iron-chelating agent often used to treat patients with acute iron poisoning, such as seen in dialysis patients with chronic renal failure. These patients are uniquely predisposed to a deadly fungal infection, called mucormycosis, because deferoxamine supplies iron that supports growth of fungi causing this infection. Apart from the important basic knowledge in delineating iron uptake mechanisms in cells, understanding how organisms causing mucormycosis obtain iron from ferrioxamine (deferoxamine bound with iron) is likely to develop strategies to treat mucormycosis infections in patients treated with deferoxamine. In this study we identified two cell surface receptors that bind ferrioxamine and facilitate iron uptake in *Rhizopus oryzae*, the most causative fungus of mucormycosis. These receptors are required for full virulence of *R*. *oryzae* in mice treated with deferoxamine. From genetic and biochemical studies it appears that the fungus binds ferrioxamine via these two receptors then liberates iron through a chemical modification step prior to transporting into the fungal cell without the internalization of deferoxamine.

## Introduction

Mucormycoses, caused by fungi in the order *Mucorales*, are life threatening infections that afflict immunosuppressed patients due to neutropenia, corticosteroids treatment, hyperglycemia, and trauma [1,2]. These relatively uncommon infections, mainly caused by *Rhizopus spp*., have been steadily increasing in numbers for the last three decades [3,4]. Despite the current aggressive treatment options against mucormycosis which constitutes reversal of immunosuppressive predisposing factors, surgical removal of infected foci (when possible) and antifungal therapy, overall mortality remains at >50% and approaches 100% for patients with brain involvement, prolonged neutropenia and hematogenously disseminated disease [5,6,7,8]. Clearly, novel strategies to prevent and/or treat the disease are critically needed.

Clinical observations strongly link the ability of organisms to obtain iron from the host as an essential virulence factor of *Mucorales* [9,10]. For example, hyperglycemia, diabetic ketoacidosis and other forms of acidosis predispose the host to mucormycosis because of compromised ability of transferrin to chelate iron, thereby making iron available to invading organisms [9,11,12]. Similarly, dialysis patients with chronic renal failure who suffer from iron overload due to blood transfusion were historically treated with the bacterial siderophore, deferoxamine [13,14,15]. These patients were uniquely susceptible to lethal form of mucormycosis [13,15,16]. Although deferoxamine is an iron-chelator from the perspective of the human host, *Rhizopus* spp. utilize ferrioxamine (deferoxamine + Fe^3+^) as a xenosiderophore to obtain previously unavailable iron [17,18]. It was also reported that *Rhizopus* obtains iron from ferrioxamine by intracellular transport of the reduced iron without deferoxamine internalization [18]. Recently, we found treatment of *Rhizopus*-infected mice with the iron chelators deferiprone [19] or deferasirox [20] (which are not utilized as xenosiderophores by *Rhizopus*) markedly improved survival. These results further confirm the unique importance of iron in the pathogenesis of mucormycosis. In the current study, we sought to identify the fungal cell surface protein that binds to ferrioxamine and its role in the pathogenesis of mucormycosis. We provide evidence that the ferrioxamine binding (Fob) cell surface proteins (namely Fob1 and Fob2) are the fungal receptors that mediate attachment to ferrioxamine, thereby facilitating fungal iron uptake from this xenosiderophore via the reductase/high affinity iron permease pathway. Importantly, Fob1 and Fob2 are inducible proteins that are required for full virulence of *R*. *oryzae* only in the deferoxamine-treated mouse model of mucormycosis.

## Results

### Identification of putative ferrioxamine receptors

**Fig 1:**
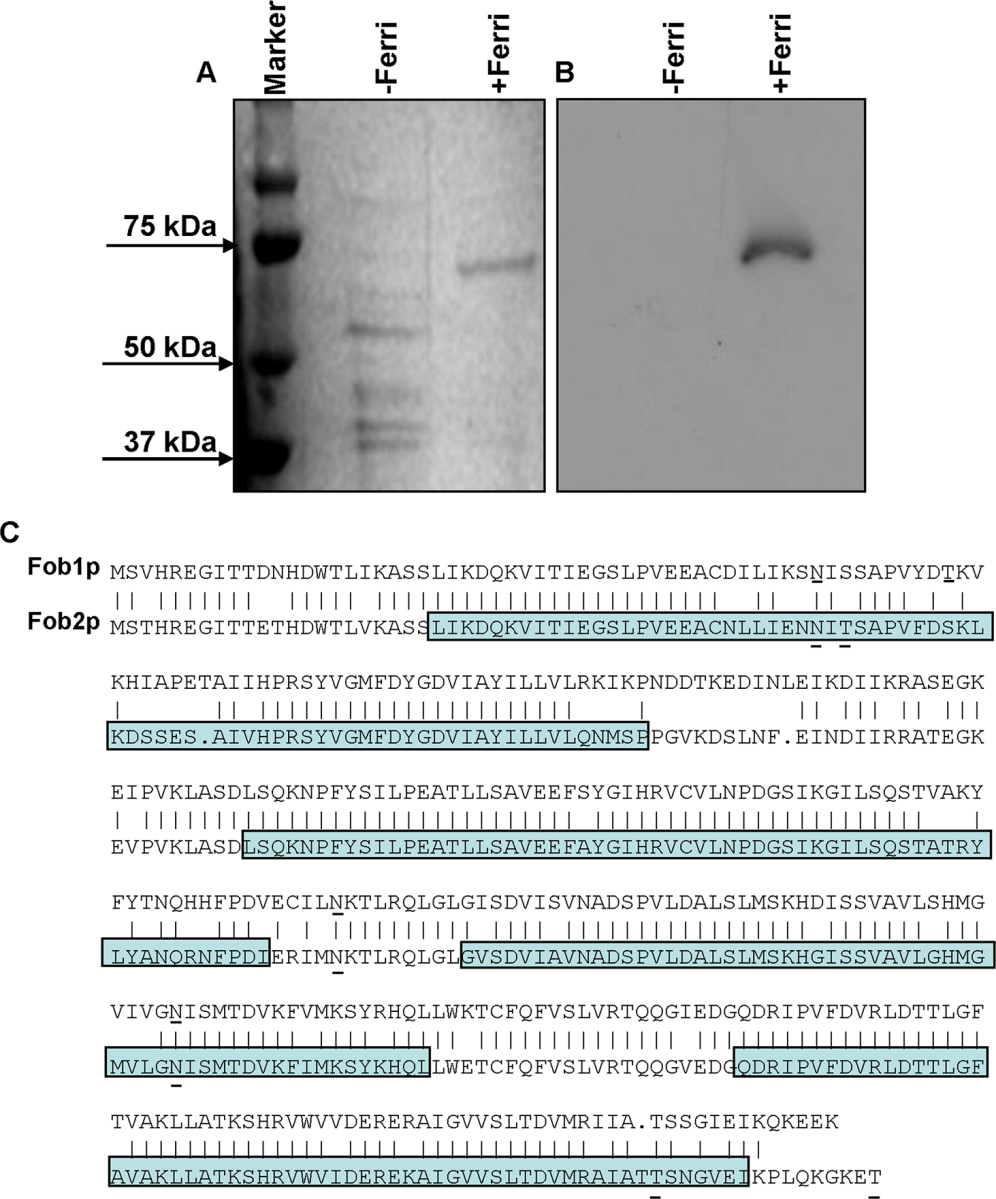
Detection of the ferrioxamine putative receptor in the cell surface material of *R*. *oryzae*. Cell surface material was collected from regenerating *R*. *oryzae* protoplasts grown in iron-limited medium with (+Ferri) or without (-Ferri) deferoxamine. Samples were separated on SDS-PAGE and visualized by Coomassie blue (A). Additionally, samples were mixed with [^55^Fe]ferrioxamine[69] then analyzed by non-denaturing PAGE followed by autoradiography (B). Two putative and closely related ORFs were identified and named *FOB1* and *FOB2*. Highlighted area denotes the four CBS domains while underlined amino acids indicate potential *N*- and *O*-glycosylation sites (C).

To isolate putative ferrioxamine receptor(s), *R*. *oryzae* protoplasts were allowed to regenerate in the presence or absence of ferrioxamine, which is the iron-rich form of the bacterial siderophore, deferoxamine [21]. SDS-PAGE analysis of proteins demonstrated the presence of a major band at ~ 70 kDa in cell-free supernatants concentrated from *R*. *oryzae* regenerating protoplasts in the presence, but not in the absence, of ferrioxamine. Other minor bands were also detected around 40kDa in supernatants from medium with or without ferrioxamine (Fig 1A). For a protein to act as a receptor it must bind to ferrioxamine. Therefore, we incubated the protein preparations collected from these supernatants with radiolabeled ferrioxamine (deferoxamine+^55^Fe) prior to running on a non-denaturing PAGE followed by autoradiography. A band from proteins concentrated in the supernatant collected from protoplasts regenerated in the presence of ferrioxamine, but not in the absence of ferrioxamine, bound to radiolabeled ferrioxamine (Fig 1B). Because the 70 kDa band was abundant in the ferrioxamine-containing supernatant and absent in the supernatant concentrated from media lacking ferrioxamine, we sequenced this band by MALDITOF MS/MS. The overwhelming indentified protein was predicted to be encoded by RO3G_11000 (352 amino acid), which is annotated as a CBS-domain-containing protein (http://www.broadinstitute.org/, see Discussion for CBS-domain protein definition) in the *R*. *oryzae* 99-880 database (also known as *R*. *delemar*) (Table 1). We also found that the ORF RO3G_11000 shares ~80% identity with ORF RO3G_05087 (350 amino acid) at the DNA or protein levels. The RO3G_05087 and RO3G_11000 ORFs are predicted to encode proteins with ~ 39 kDa in size and were named *FOB1*, and *FOB2*, respectively. These predicted proteins have 4 putative CBS domains and multiple possible *N-* and *O-*glycosylation sites (Fig 1C). Interestingly and despite the presence of Fob2 protein in the supernatant of regenerated protoplasts, we did not observe classic features of secreted proteins such as N-terminal signal peptide or C-terminus GPI-anchor sequence. However, using the MEMSAT program it was predicted that Fob1 and Fob2 proteins have one to two transmembrane domains with an extracellular fragment and a cytoplasmic tail (S1 Fig). Further localization analysis using antibody staining confirmed the surface localization of Fob proteins (see below).

**Table 1:** MALDITOF MS/MS sequence results of the 70 kDa band detected in the supernatant of regenerated protoplasts of *R*. *oryzae* grown in the presence of ferrioxamine.

Rank order	Protein	Protein mass (kDa)	Protein score	Queries matched
1	RO3G_11000 (Fob2 protein)	38.9	55	15
2	RO3G_00635 (Hypothetical protein)	94	33	13
3	RO3G_04771 (Hypothetical protein)	78	30	6
4	RO3G_03963 (Hypothetical protein)	98.8	30	5
5	RO3G_01777 (phosphoribosyl-glycinamide formyltransferase)	22.7	30	4
6	RO3G_01375 (Hypothetical protein)	40.9	30	4

Protein scores of >29 was calculated for the 5% confidence threshold.

We also examined whether this family of genes was present in other *Mucorales* known to cause mucormycosis. Blast search of *FOB1* and *FOB2* confirmed the presence of orthologs of *FOB1/2* in every *Mucorales* genome published thus far with percent identity at the amino acid levels ranging from 42% for *Mortierella verticillata FOB1* ortholog to 78% for *Mucor circinelloides f*. *circinelloides FOB2* (Table 2). Orthologs from other fungi were also found with lesser degree of identity (e.g. ~ 20% for *Aspergillus*, *Saccharomyces cerevisiae*, and *Candida*).

**Table 2:** Sequence homology of *FOB1/2* genes from *R*. *oryzae* 99-880 to possible *Mucorales* orthologs.

Strain ID	*Mucorales*	Source	% ID *FOB1*	% ID *FOB2*
JMRC:FSU:96821	*Lichtheimia corymbifera*	Jena Uni	ND	59 (355)
JMRC FSU:6197	*Lichtheimia ramosa*	Jena Uni	ND	58 (355)
NRRL 6337	*Mortierella verticillata*	Broad Inst.	42 (327)	45 (327)
1006 PhL	*Mucor circinelloides f*. *circinelloides*	Broad Inst.	57 (330)	78 (356)
CBS 183.67	*Rhizomucor pusillus*	Genozymes	ND	59 (346)

Numbers in brackets represent the orthologs length in amino acids.

% ID denotes % identity.

ND indicate not detected.

### Putative ferrioxamine receptors are inducible by ferrioxamine, likely to oligomerize, and expressed on the cell surface

**Fig 2:**
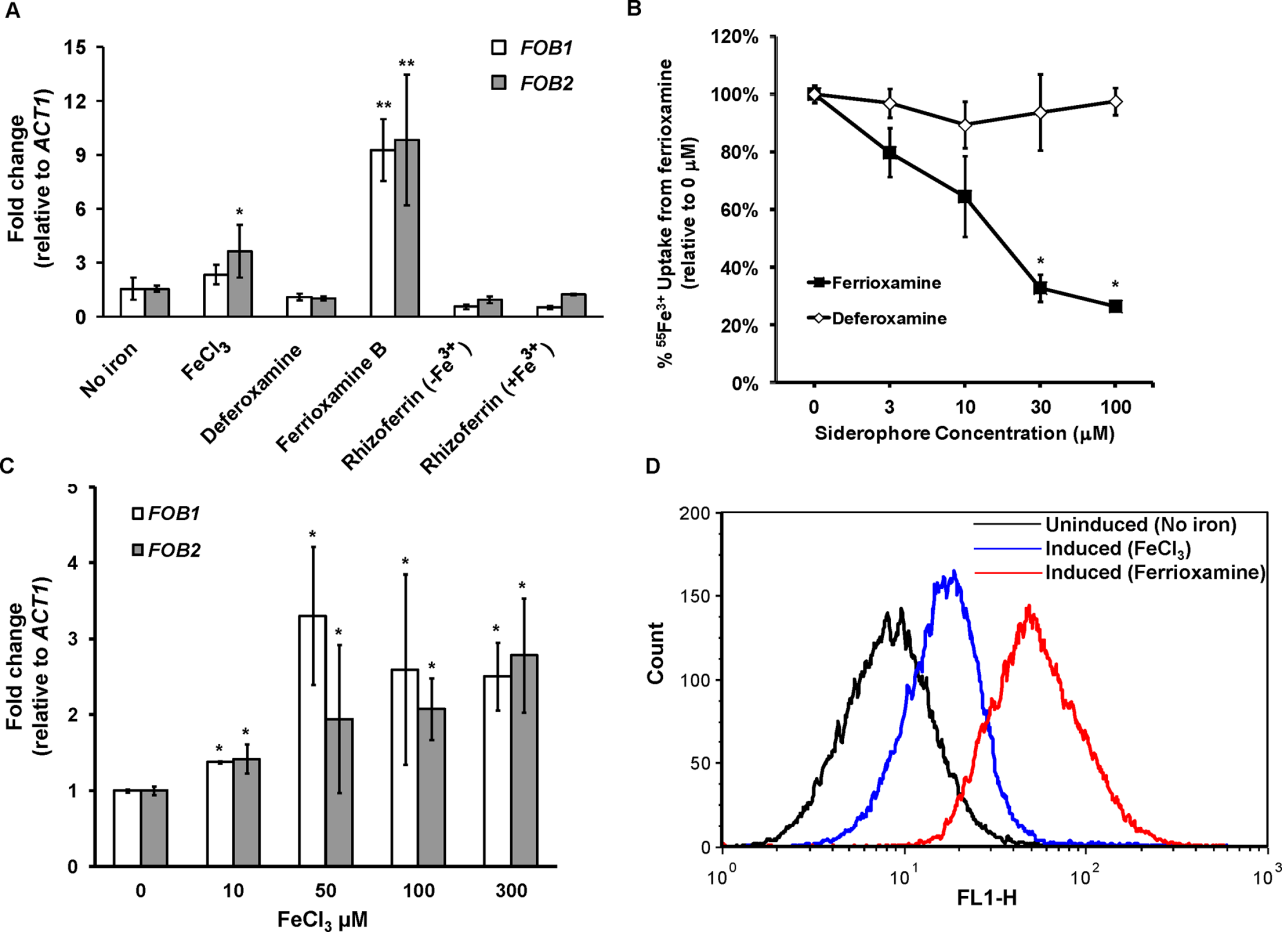
*FOB1* and *FOB2* expression is induced by ferrioxamine and to a lesser extent by ferric iron but not by deferoxamine. The expression of *FOB1* and *FOB2* was studied in response to iron sources *in vitro*. (A) qRT-PCR was used to study the expression of *FOB1* and *FOB2* in media without added iron, 10 μM of ferrioxamine, deferoxamine, rhizoferrin without Fe^3+^, rhizoferrin with Fe^3+^ or 100 μM FeCl_3_. (B) Cold ferrioxamine but not deferoxamine inhibits ^55^Fe^3+^ uptake from ferrioxamine (introduced at a concentration of 10 μM). (C) Compared to ferrioxamine, FeCl_3_ demonstrated modest and non-linear increase in *FOB1* and *FOB2* expression as determined by qRT-PCR. (D) Flow cytometry using antiFob2p polyclonal antibodies demonstrated the cell surface expression of Fob1 and Fob2 proteins in medium containing ferrioxamine but not in medium without iron supplement. Error bars represent the standard deviation of the mean from at least two independent assays (n> 6 per group). *P<0.05 vs. no iron, while **P<0.001 vs. no iron or FeCl_3_.

Because radiolabeled ferrioxamine bound to a band in protein preparations from supernatants collected from regenerated protoplasts in the presence of ferrioxamine and not in the siderophore’s absence (Fig 1B), we reasoned that the ferrioxamine receptor is likely induced by ferrioxamine. To test if *FOB2* and its *FOB1* homologue fulfill this criterion, we cultured *R*. *oryzae* 99-880 in medium containing ferrioxamine, FeCl_3_, deferoxamine, rhizoferrin (a siderophore produced by *Rhizopus* [18,22]), or Fe^3+^ containing rhizoferrin as the sole source of iron prior to analyzing the expression of *FOB1* and *FOB2* by qRT-PCR. As can be seen in Fig 2A, only the iron-rich ferrioxamine consistently enhanced the expression of *FOB1* and *FOB2* by ~ 9 fold vs. any other condition. Neither the iron depleted ferrioxamine (i.e. deferoxamine), nor rhizoferrin with or without iron induced the expression of either *FOB1* or *FOB2*. However, the presence of FeCl_3_ as a source of iron instead of ferrioxamine resulted in a modest increase in the expression of *FOB2*.

If *FOB2* and/or *FOB1* indeed encode a ferrioxamine receptor, the lack of their expression by deferoxamine indicated that iron uptake from radiolabeled ferrioxamine should not be inhibited by deferoxamine but rather by cold ferrioxamine. To test this possibility, iron uptake of ^55^Fe^3+^ from ferrioxamine by *R*. *oryzae* was carried out at increased concentrations of cold ferrioxamine or deferoxamine. Cold ferrioxamine, but not deferoxamine, inhibited ^55^Fe^3+^ uptake by >70% when introduced at 3–10 fold higher concentrations than the radiolabeled ferrioxamine (Fig 2B).

Because FeCl_3_ had a modest effect on the expression of *FOB1* and *FOB2* when compared to ferrioxamine, while deferoxamine had no effect on their expression, this implicated ferric iron as a critical signal for triggering the expression of these two genes. We studied the expression of *FOB1* and *FOB2* in response to varying concentrations of FeCl_3_. As can be seen in Fig 2C, FeCl_3,_ modestly enhanced the expression of both genes in a non-linear manner. Collectively, these results show that the expression of *FOB1* and *FOB2* is maximal only when the siderophore is iron–rich (i.e. ferrioxamine), the iron-depleted form (i.e. deferoxamine) does not induce nor it inhibits iron uptake from ferrioxamine, and the native siderophore of rhizoferrin does not induce the expression of these two genes.

**Fig 3:**
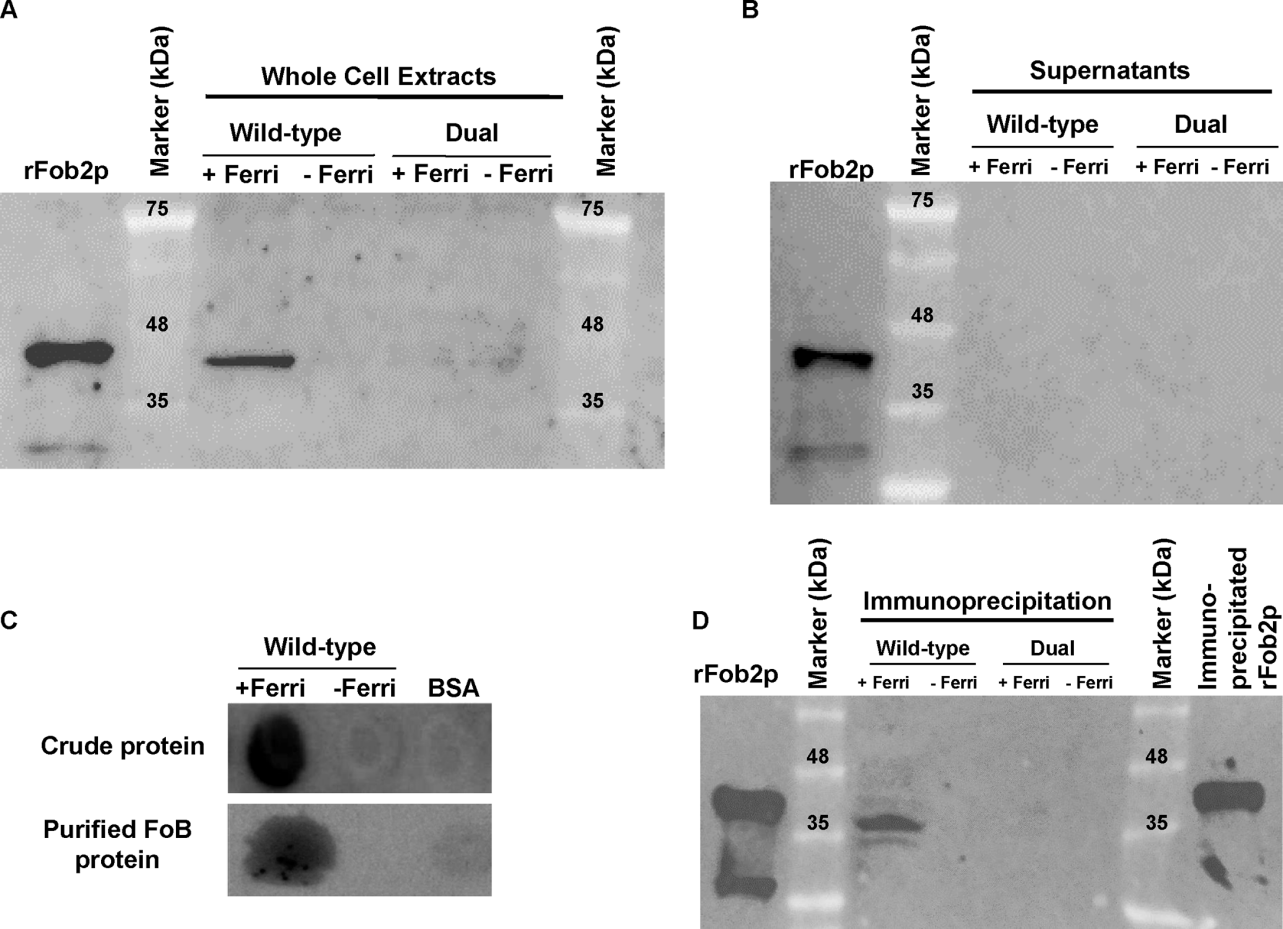
Western blot analysis demonstrating that Fob proteins are cell-associated, not secreted, and bind to radiolabeled ferrioxamine. Antibodies were raised against *E*. *coli-*produced Fob2 protein and used to study the expression pattern of Fob proteins. (A) Western blot analysis of whole cell proteins extracted from *R*. *oryzae* wild-type or *R*. *oryzae* mutant with dual inhibition of *FOB1/ FOB2* by RNA-i (see Fig 6 for generation of this mutant). (B) Western blot analysis of supernatant (cell-free) collected from *R*. *oryzae* wild-type hyphae or *R*. *oryzae* mutant with dual inhibition of *FOB1/ FOB2* growing for overnight in media with or without ferrioxamine. (C) Whole cell proteins from *R*. *oryzae* wild-type grown in medium with or without ferrioxamine were loaded on membrane under non-denaturing conditions prior to mixing with [^55^Fe]ferrioxamine[69] then analyzed with autoradiography. Also, Fob proteins from the same crude cell wall extracts were separated by immunoprecipitation using anti-IgG Fob2 antibodies coupled to protein G beads column prior to incubating with [^55^Fe]ferrioxamine under non-denaturing conditions. The beads were washed extensively with washing buffer prior to spotting them on membrane followed by autoradiography. (D) Western blot analysis of whole cell proteins extracted from *R*. *oryzae* wild-type or *R*. *oryzae* mutant with dual inhibition of *FOB1/ FOB2* following immunoprecipitation using anti-IgG Fob2 antibodies as in C. rFob2p was included in A, B, and D as a control showing the expected band size of ~40 kDa (bands in A, B and D below 35 kDa from rFob2p lane represent degradation of rFob2p. Note, that the marker is overlaid on each of the western blots to determine band sizes.

To study if enhancement in *FOB1* and *FOB2* gene expression resulted in enhanced protein expression on *R*. *oryzae* cell surface, we raised antibodies against an *E*. *coli*-produced Fob2 protein (rFob2p) by vaccinating mice and used these polyclonal antibodies to detect the expression of Fob proteins on *R*. *oryzae* cell surface by FACS analysis. Sera collected from mice vaccinated with rFob2p + adjuvant had an antibody titer of 1:32,000 vs. a titer of 1:1600 of sera collected from mice vaccinated with the adjuvant alone. Additionally, sera collected from rFob2p vaccinated mice reacted to the *E*. *coli* produced Fob2 protein as well as a protein band isolated from whole cell protein extract of *R*. *oryzae* wild-type hyphae only when grown in the presence of ferrioxamine. These two bands were detected at ~40 kDa with slight difference in size which is likely attributed to the presence of His-tag on the rFob2p. Also the antibodies did not detect any bands from whole cell extracts of *R*. *oryzae* strain in which both *FOB1* and *FOB2* were down regulated regardless of the presence or absence of ferrioxamine in the growth medium (see below for *FOB1/FOB2* inhibition by RNA-i) (Fig 3A for Western blot and S2A Fig for SDS-PAGE gel picture). Analysis of the recognized bands by nano-LC ESI MS/MS confirmed the identity of the proteins to Fob2p (Table 3). Interestingly, we could not detect the protein in cell-free supernatants collected from media containing *R*. *oryzae* wild-type or *FOB1/FOB2* inhibition mutant grown in the presence or absence of ferrioxamine (Fig 3B for Western blot, and S2B Fig for SDS-PAGE gel picture). Furthermore, radiolabeled ferrioxamine reacted to crude cell protein extracts or proteins immunoprecipitated by anti-Fob2 antibodies from *R*. *oryzae* wild-type whole cell extracts only when grown in a medium containing ferrioxamine (Fig 3C). Finally, the immunoprecipitated band from the whole cell extract of *R*. *oryzae* wild-type cells as well as the immunoprecipitated band from rFob2p had sizes of around 40 kDa (Fig 3D for Western blot and S2C Fig for SDS-PAGE gel picture) similar to what noticed in Fig 3A. These two immunoprecipitated bands were confirmed to be Fob2 protein by nano-LC ESI MS/MS (Table 3). Because immunoprecipitation of whole cell extracts of *R*. *oryzae* wild-type cells resulted in almost no apparent band on SDS-PAGE gel, we reasoned that the majority of the protein was retained bound to the antibody coated beads. Therefore, we ran the beads on a SDS-PAGE gel after boiling them. Coomassie Brilliant Blue stain showed two major bands at ~40 kDa as well as ~70 kDa (S2D Fig). Because the 70 kDa band corresponded to the size of the identified Fob2 protein from concentrated supernatant of regenerated protoplasts grown in ferrioxamine-containing medium (Fig 1A), we sequenced this band as well as the 40 kDa band by nano-LC ESI MS/MS. The major protein present in these two bands was identified as Fob2p (Table 3). Collectively, these results demonstrated the specificity of the antibodies to Fob proteins (the antibodies are likely to recognize Fob1 and Fob2 proteins due to the predicted similar size and the 80% identity), strongly indicate that Fob proteins are cell-associated rather than secreted, and likely Fob2 proteins oligomerize under certain conditions to form larger protein size.

**Table 3:** nano-LC ESI MS/MS sequence data of 40 or 70 kDa bands from whole cell extracts of *R*. *oryzae* wild-type before and after immunoprecipitation.

Accession	Description	Σ# Unique Peptides	Band 1	Band 2	Band 3	Band 4	Band 5	Amino acid #	MW [kDa]	Calc. pI
I1CCV9	Uncharacterized protein *R*. *oryzae* RO3G_11000 (Fob2p)	22	3.6E9	2.5E9	4.2E9	5.1E8	1.4E9	351	38.7	6.37
I1BY51	Glyceraldehyde-3-phosphate dehydrogenase 3 *R*. *oryzae* RO3G_05836	9	0	6.7E6	0	0	1.3E9	338	36.1	6.16
I1CEK5	Transaldolase *R*. *oryzae* RO3G_11596	14	0	2.4E7	0	0	3.8E9	322	35.7	5.40
I1BPM3	Uncharacterized protein *R*. *oryzae* RO3G_02857	7	0	0	0	0	1.2E8	286	30.9	6.07
I1CJ47	Glyceraldehyde-3-phosphate dehydrogenase 1 *R*. *oryzae* RO3G_13188	7	0	0	0	0	5.4E8	333	35.6	7.84
I1C0C1	Uncharacterized protein *R*. *oryzae* RO3G_06606	6	0	0	0	0	1.9E7	259	28.0	5.54
I1C4E8	Glyceraldehyde-3-phosphate dehydrogenase 2 *R*. *oryzae* RO3G_08033	5	0	8.6E6	0	0	8.5E8	338	35.9	6.57
I1BW63	UDP-glucose 4-epimerase *R*. *oryzae* RO3G_05148	7	0	0	0	0	4.0E7	354	38.6	6.55
I1CCY5	Saccharopine dehydrogenase R. oryzae RO3G_11026	9	0	0	0	0	1.7E8	451	49.3	5.66
I1C642	Uncharacterized protein *R*. *oryzae* RO3G_08627	6	0	0	0	0	1.9E7	364	40.3	5.69
I1CLC6	Endopeptidase *R*. *oryzae* RO3G_13967	9	1.9E6	1.7E7	1.3E8	0	1.5E9	401	44.4	4.87
I1BQ83	3-isopropylmalate dehydrogenase *R*. *oryzae* RO3G_03067	7	0	0	0	0	2.1E7	374	39.8	5.41
I1CD60	Uncharacterized protein *R*. *oryzae* RO3G_11101	5	0	0	0	0	3.3E7	312	34.9	6.64
I1C3R1	Uncharacterized protein *R*. *oryzae* RO3G_07796	8	0	0	2.1E7	0	2.2E7	579	63.6	6.24
I1BYI4	Uncharacterized protein *R*. *oryzae* RO3G_05969	3	0	0	1.3E7	0	6.7E6	238	26.9	6.52
I1CLG1	Actin-2 *R*. *oryzae* RO3G_12558	3	9.3E7	1.3E8	7.6E7	4.630E7	3.6E7	375	41.7	5.58
I1BPT0	Transaldolase *R*. *oryzae* RO3G_02914	1	0	2.2E7	0	0	1.4E9	322	35.9	7.11
I1CKV3	ADP-ribosylation factor 1 *R*. *oryzae* RO3G_13794	1	4.4E7	0	0	0	0	163	18.7	6.02
I1C2J3	Glucosamine-6-phosphate isomerase 1 *R*. *oryzae* RO3G_07378	1	0	0	0	0	0	298	33.4	6.48
I1C4Y4	Histone H4 *R*. *oryzae* RO3G_08122	1	1.8E7	1.3E7	0	0	0	103	11.4	11.36
I1BZG8	Uncharacterized protein R. oryzae RO3G_06303	1	0	0	0	0	0	304	34.0	6.19
I1BVE5	Acetyl-CoA hydrolase R. oryzae RO3G_04880	1	0	0	0	0	0	524	58.3	6.49
I1BTR0	Uncharacterized protein *R*. *oryzae* RO3G_04295	2	0	0	0	0	1.3E7	421	45.8	4.15
I1CE02	Cyclin domain-containing protein *R*. *oryzae* RO3G_11393	1	0	0	0	2.889E10	0	101	11.7	5.91
I1CEM3	Uncharacterized protein *R*. *oryzae* RO3G_11614	1	0	0	0	0	9.4E6	162	18.4	6.18
I1BU17	Uncharacterized protein *R*. *oryzae* RO3G_04402	2	0	0	0	5.672E6	7.4E6	510	57.7	5.17
I1BPG1	Uncharacterized protein *R*. *oryzae* RO3G_02795	2	0	0	0	0	4.2E6	543	58.8	5.73
I1BUG8	Uncharacterized protein *R*. *oryzae* RO3G_04553	1	0	0	0	0	6.4E6	460	40.8	3.81
I1BKQ1	Chitin deacetylase *R*. *oryzae* RO3G_01485	2	0	0	1.917E6	2.090E6	5.4E6	416	45.8	4.70
I1BLY8	Uncharacterized protein *R*. *oryzae* RO3G_01922	1	0	0	0	0	4.4E6	485	45.6	3.65
I1BN63	Uncharacterized protein *R*. *oryzae* RO3G_02347	1	0	0	0	0	1.3E7	386	42.3	9.19
I1BQ37	Uncharacterized protein *R*. *oryzae* RO3G_03021	1	0	0	0	0	2.2E7	720	83.0	5.20
I1C8H3	Elongation factor Ts, mitochondrial *R*. *oryzae* TSF1	1	0	0	0	0	7.1E6	331	35.9	6.02
I1BRN0	Uncharacterized protein *R*. *oryzae* RO3G_03565	1	0	2.150E6	0	0	0	333	34.2	11.87
I1CDZ8	Uncharacterized protein *R*. *oryzae* RO3G_11389	1	0	0	0	0	6.7E6	423	46.7	4.54
I1C1Z0	Uncharacterized protein *R*. *oryzae* RO3G_07175	1	0	0	1.950E6	8.944E5	0	414	47.8	6.90
I1CDD0	Uncharacterized protein *R*. *oryzae* RO3G_11171	1	0	0	7.562E6	0	0	438	49.8	5.45
I1C5V4	Uncharacterized protein *R*. *oryzae* RO3G_08539	1	0	0	0	0	1.0E6	608	67.7	5.59
I1CM95	Uncharacterized protein *R*. *oryzae* RO3G_14286	1	0	0	0	0	1.0E8	773	87.1	6.25
I1BP17	Uncharacterized protein *R*. *oryzae* RO3G_02651	1	0	0	0	2.295E6	0	615	69.3	5.25
I1BLJ2	Pyruvate decarboxylase isozyme *R*. *oryzae* RO3G_01776	1	0	0	0	0	3.2E6	560	61.3	5.45
I1BU19	Uncharacterized protein *R*. *oryzae* RO3G_04404	1	0	0	0	0	1.5E6	614	67.9	6.27
I1CNE5	Uncharacterized protein *R*. *oryzae* RO3G_14686	1	0	0	2.832E6	0	0	877	100.3	6.83
I1BKY8	Uncharacterized protein *R*. *oryzae* RO3G_01572	1	0	0	0	0	0	700	77.5	7.30
I1BNP9	Uncharacterized protein *R*. *oryzae* RO3G_02533	1	0	0	0	0	2.0E6	680	75.0	5.85

Band 1, rFob2p; Band 2, 40 kDa band of wild-type whole cell extract after immunoprecipitation; Band 3, 40 kDa band from beads coated with antiFob2 antibodies and used for immunoprecipitation of wild-type whole cell extract; Band 4, 70 kDa band from beads coated with antiFob2 antibodies and used for immunoprecipitation of wild-type whole cell extract; Band 5, 40 kDa band of wild-type whole cell extract without immunoprecipitation; Calc. pI, calculated isoelectric point.

To discern if the proteins are expressed on the cell surface or intracellularly, we used the anti-Fob2p antibodies to stain *R*. *oryzae* germlings without permeabilization and studied the staining patterns by flow cytometry and confocal imaging. We noticed enhanced fluorescence when *R*. *oryzae* wild-type cells were incubated with ferrioxamine and to a lesser extent when FeCl_3_ was used as the sole source of iron compared to fungal cells grown in the absence of iron source (Fig 2D). Confocal images using these antibodies also confirmed the cell surface localization of Fob proteins when the fungal germlings were incubated with ferrioxamine (S3 Fig). Collectively, these results show that Fob proteins are expressed on the cell surface of germinated *R*. *oryzae* when ferrioxamine is present.

### *FOB1* or *FOB2* gene silencing by RNA-i modestly reduces iron uptake and slows growth of *R*. *oryzae* on medium supplemented with ferrioxamine

**Fig 4:**
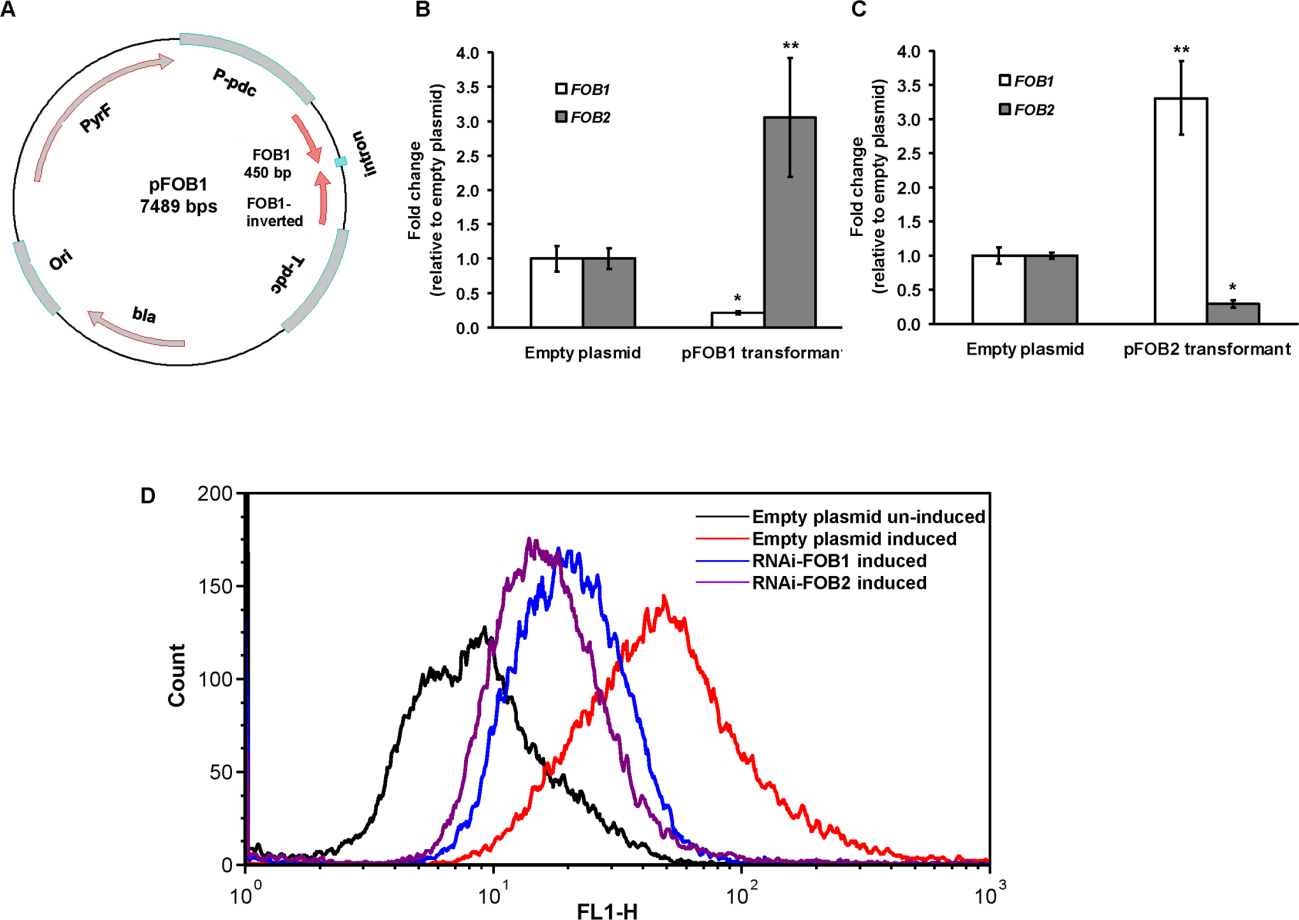
RNA-i targeting a single putative ferrioxamine receptor leads to compensatory effect with the other receptor. Plasmid pFOB1 (or pFOB2) targeting inhibition of a single putative ferrioxamine receptor (A). Plasmid pFOB1 (B) or pFOB2 (C) successfully inhibited the expression of *FOB1* and *FOB2*, respectively as determined by qRT-PCR. However, a compensatory gene expression was observed in these transformants from the untargeted gene. Results (n = 6 per group) were expressed as fold change in gene expression relative to gene expression in empty plasmid transformant. Error bars represent the standard deviation of the mean from two independent assays. * and **P<0.05 compared to *FOB1* and *FOB2* expression in empty plasmid transformant, respectively. Flow cytometry using antiFob2 polyclonal antibodies confirmed cell surface expression and the compensatory effect of the untargeted gene. Induction and un-induction conditions were carried out using YNB with or without 10 μM ferrioxamine (D).

To determine the role of these putative receptors in the uptake of iron from ferrioxamine, we down regulated the expression of either of *FOB1* or *FOB2* (Fig 4A). As expected RNA-i constructs targeting either of the 2 ORFs resulted in ~90% inhibition of the expression of the intended target (i.e., *FOB1* or *FOB2*). However, there was no inhibition in the expression of the other gene (Fig 4B and 4C). In contrast, gene silencing of one *FOB* appeared to induce the expression of the other gene (e.g., a plasmid targeting *FOB1* blocked expression of *FOB1* expression, while *FOB2* expression increased compared to cells transformed with empty plasmid and grown in the presence of ferrioxamine) (Fig 4B and 4C). These findings were further confirmed by immunostaining and FACS analysis. Cell surface expression of *R*. *oryzae* transformed with RNA-i constructs targeting either *FOB1* or *FOB2* had reduced levels compared to *R*. *oryzae* cells transformed with empty plasmid and grown in the presence of ferrioxamine. However, this reduction in Fob protein expression did not reach the low levels of *R*. *oryzae* transformed with the empty plasmid and grown in the absence of ferrioxamine (Fig 4D). These results indicate that targeting one *FOB* gene results in a gene expression compensatory effect of the other.

**Fig 5:**
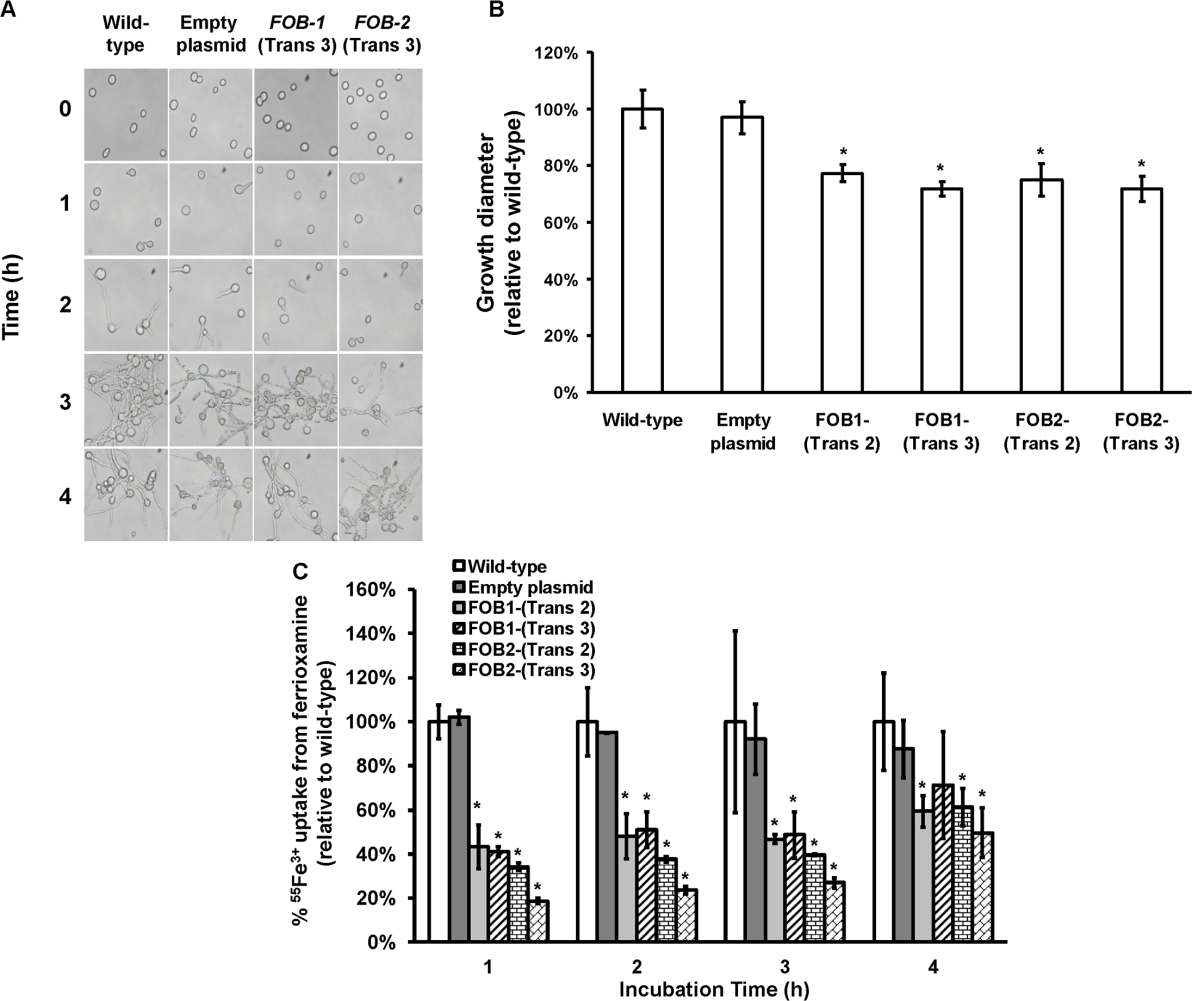
Single gene inhibition of *FOB1* or *FOB2* slightly reduced germination, growth and iron uptake of *R*. *oryzae* when ferrioxamine is used as a sole source of iron. (A) To determine effect of gene inhibition on *R*. *oryzae* germination, cells were pregerminated in YNB+CSM-URA medium supplemented with 10 μM ferrioxamine as a sole source of iron. At selected time intervals samples were taken from the medium and examined by light microscopy. (B) To determine effect of gene inhibition on growth of *R*. *oryzae*, 10^5^ spores of each strain were plated in the middle of the YNB+CSM-URA agar plates supplemented with 10 μM ferrioxamine as a sole source of iron and the colony diameter (mm) was measured. Two independent transformants from each construct were tested in triplicate (n = 9). *P <0.05 vs. wild-type and empty plasmid strains. (C) Single gene inhibition attenuated ^55^Fe^3+^ from ferrioxamine. Two independent transformants from each construct were tested in triplicate (n = 9). *P <0.05 vs. Wild-type and empty plasmid strains. In B and C, error bars represent the standard deviation of the mean from three independent assays.

Having generated the two *R*. *oryzae* strains with reduced expression in *FOB1* or *FOB2*, we compared the *FOB1* or *FOB2* silencing effect on the germination and growth of *R*. *oryzae* in a medium supplemented with ferrioxamine. Silencing *FOB1* or *FOB2* did not have an effect on the ability of *R*. *oryzae* to germinate in a medium supplemented with ferrioxamine (Fig 5A). In contrast, *R*. *oryzae* transformed with RNA-i constructs targeting *FOB1* or *FOB2* gene expression resulted in 20–25% inhibition in growth on medium supplemented with ferrioxamine as a sole source of iron when compared to *R*. *oryzae* wild-type or *R*. *oryzae* strain transformed with the empty plasmid (Fig 5B). This reduction in growth is likely due to the compromised ability of *R*. *oryzae* transformed with RNA-i constructs, targeting *FOB1* or *FOB2*, to take up iron from ferrioxamine since attenuation of either *FOB1* or *FOB2* reduced the ability of *R*. *oryzae* to take up iron from radiolabeled ferrioxamine (Fig 5C). Although initial incubation periods of 1–2 hrs showed 60–80% reduction in ^55^Fe^3+^ uptake from ferrioxamine among *R*. *oryzae* transformed with either RNA-i constructs targeting *FOB1* or *FOB2* compared to wild-type or *R*. *oryzae* transformed with empty plasmid, only 45% inhibition was detected after 4 hrs. This modest reduction in iron uptake is likely due to the compensatory expression of the other *FOB* gene.

### Gene silencing of both *FOB1* and *FOB2* affects germination and drastically reduces growth and iron uptake from ferrioxamine

**Fig 6:**
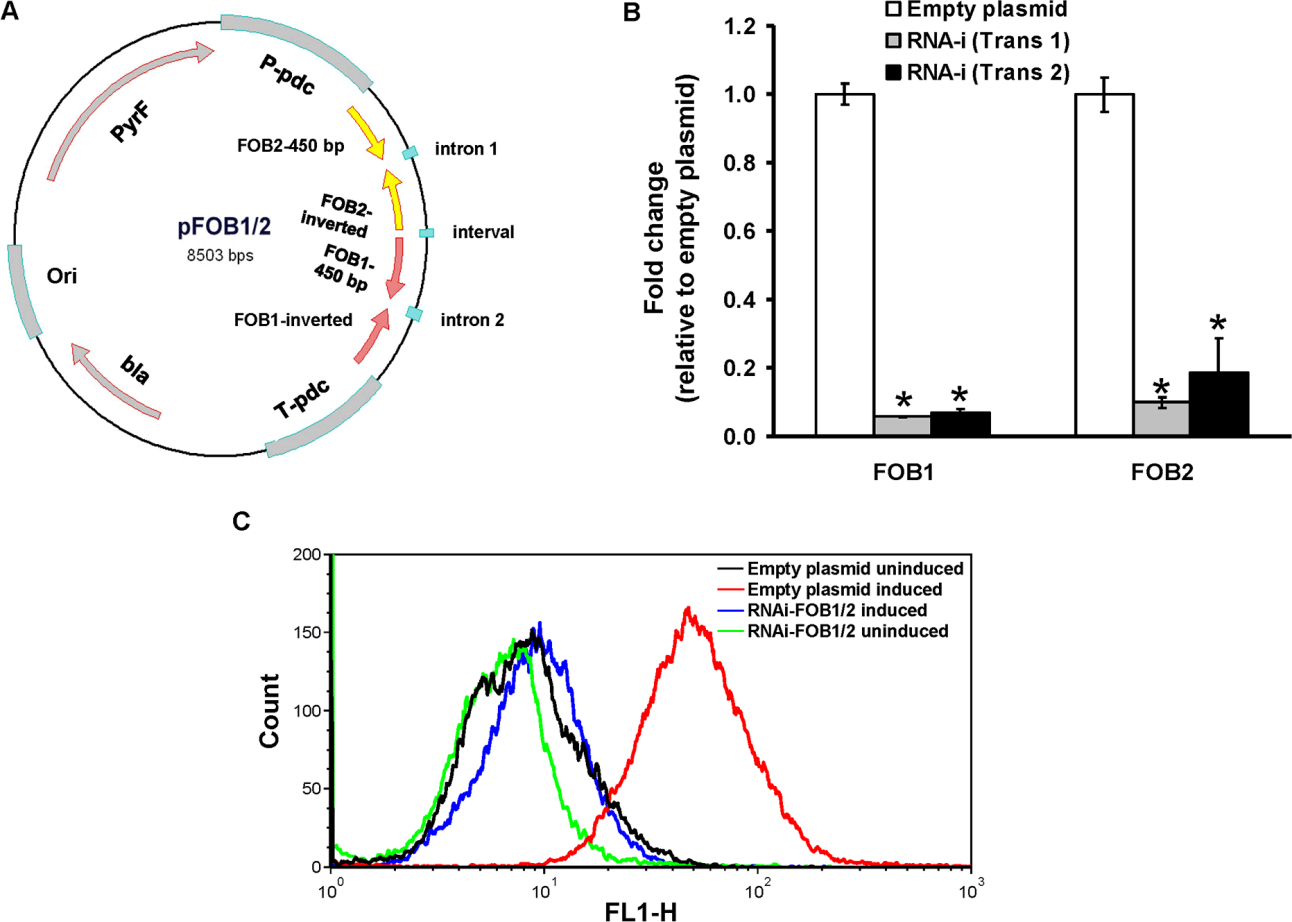
Dual gene inhibition strategy completely abrogates *FOB1* and *FOB2* expression. Plasmid pFOB1/FOB2 targeting inhibition of the dual putative ferrioxamine receptors (A). Plasmid pFOB1/FOB2 almost completely blocked *FOB1* and *FOB2*, respectively as determined by qRT-PCR (B). Results were expressed as fold change in gene expression relative to empty plasmid transformant. Error bars represent the standard deviation of the mean from two independent assays. *P<0.005 compared to empty plasmid transformant. Flow cytometry using antiFob2 polyclonal antibodies confirmed the lack of expression of either Fob1 or Fob2 proteins on the cell surface of *R*. *oryzae* transformed with pFOB1/FOB2 relative to cells transformed with empty plasmid (C). Induction and un-induction conditions were carried out using YNB with or without 10 μM ferrioxamine.

**Fig 7:**
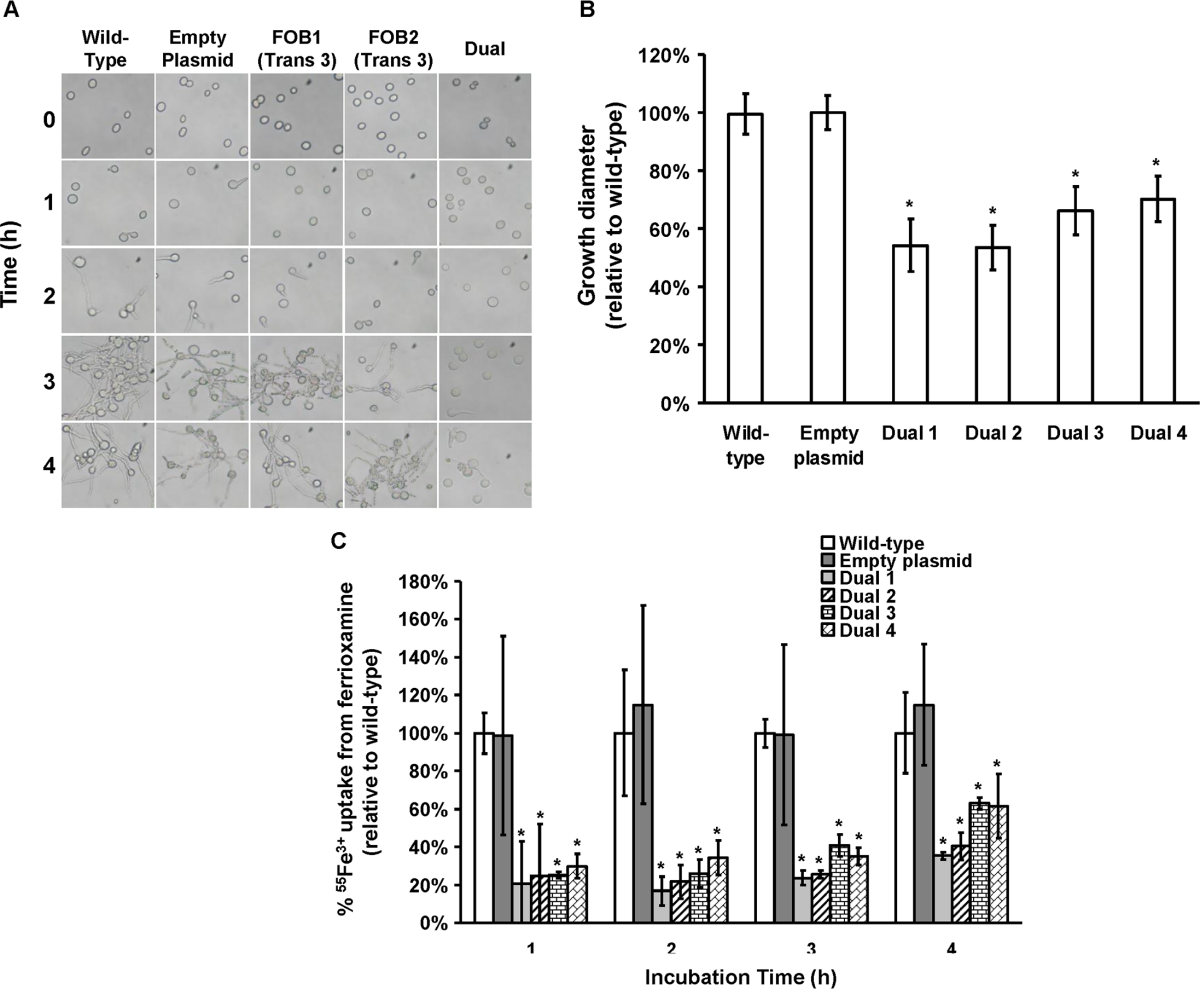
Dual gene inhibition significantly attenuated the ability of *R. oryzae* to germinate, grow and take up iron from ferrioxamine. (A) To determine effect of dual gene inhibition on *R*. *oryzae* germination, cells were pregerminated in YNB+CSM-URA medium supplemented with 10 μM ferrioxamine as a sole source of iron. At selected time intervals samples were taken from the medium and examined by light microscopy. (B) To determine effect of dual gene inhibition on growth of *R*. *oryzae*, 10^5^ spores of each strain were plated in the middle of the YNB+CSM-URA agar plates supplemented with 10 μM ferrioxamine as a sole source of iron and the colony diameter (mm) was measured. Four independent transformants were tested in triplicate (n = 9). *P <0.01 vs. wild-type and empty plasmid strains. (C) Dual gene inhibition attenuated ^55^Fe^3+^ from ferrioxamine. Four independent transformants were tested in triplicate (n = 9). *P <0.05 vs. wild-type or empty plasmid strains. In B and C, error bars represent the standard deviation of the mean from three independent assays.

Because gene silencing of one of the two *FOB* genes resulted in a modest effect on growth and iron uptake from ferrioxamine, we employed a strategy of silencing both genes in a single construct driven by a single *pdc* promoter (i.e., pFOB1/2) (Fig 6A). Upon transforming *R*. *oryzae* with this construct we were able to reduce the expression of *FOB1* and *FOB2* by >90% (Fig 6B). Additionally, anti-Fob2p antibodies could not detect any bands from whole cell protein extract of *R*. *oryzae* transformed with dual inhibition construct and grown in the presence or absence of ferrioxamine (Fig 3A). Moreover, the inhibition of *FOB1/FOB2* translated to near complete blocking of cell surface expression to the level of *R*. *oryzae* transformed with the empty plasmid and grown in the absence of ferrioxamine (Figs 6C and S3). Furthermore, the dual inhibition of *FOB1* and *FOB2* resulted in severe retardation of *R*. *oryzae* germination (Fig 7A) and more than 40–50% inhibition in growth on medium supplemented with ferrioxamine (Fig 7B) when compared to wild-type strain or *R*. *oryzae* transformed with empty plasmid. Finally, this retardation in germination and growth on ferrioxamine supplemented medium was accompanied by up to 85% inhibition of iron uptake from ferrioxamine (Fig 7C). Notably, there was no difference in growth of any of the three strains on FeCl_3_ supplemented YNB medium (S4 Fig). Collectively, these results clearly implicate Fob1 and Fob2 proteins in the uptake of iron from ferrioxamine.

### *FOB1* and *FOB2* are required for full virulence of *R*. *oryzae* in the deferoxamine-treated but not the diabetic ketoacidotic mouse models

**Fig 8:**
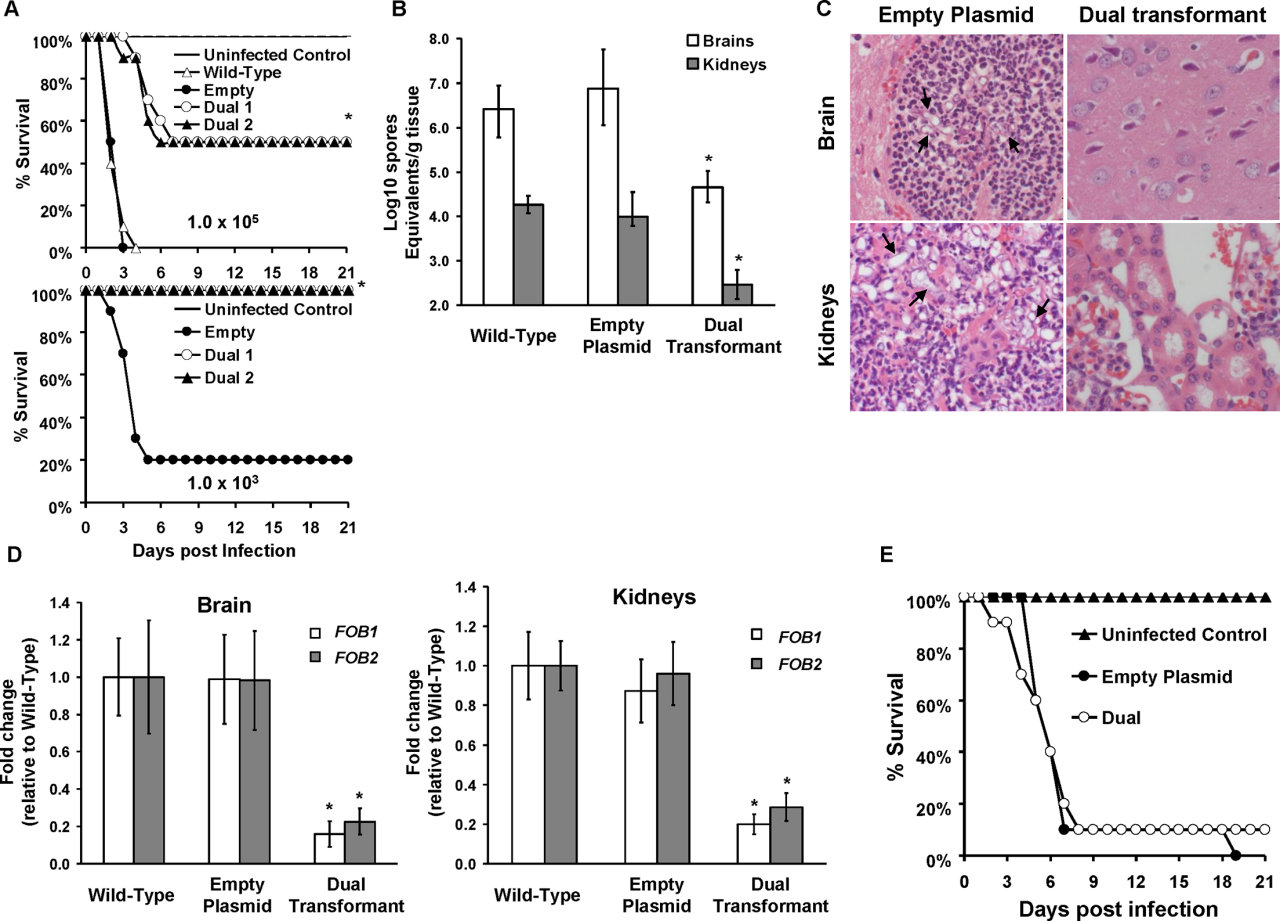
*FOB1* and *FOB2* are required for the full virulence of *R*. *oryzae* in the deferoxamine-treated and not the DKA mouse models. (A) Survival of mice (n = 10 per arm) treated with three doses of deferoxamine (100 mg/kg/dose) via i.p. injection on day -1, 0, and +1 relative to intravenous infection with *R*. *oryzae* strains. For the targeted inoculum of 1 x 10^5^, mice were infected with wild-type (6.5 x 10^4^ spores), *R*. *oryzae* transformed with empty plasmid (8.6 x 10^4^ spores) or *R*. *oryzae* with dual attenuated expression of *FOB1* and *FOB2* (Dual 1 at 4.6 x 10^4^ spores or Dual 2 at 3.6 x 10^4^ spores). For the targeted inoculum of 1 x 10^3^, mice were infected with *R*. *oryzae* transformed with the empty plasmid (9.5 x 10^2^ spores) or *R*. *oryzae* with dual attenuated expression of *FOB1* and *FOB2* (Dual 1 at 7.9 x 10^2^ spores or Dual 2 at 4.9 x 10^2^ spores). *P<0.001 compared to mice infected with wild-type or *R*. *oryzae* transformed with empty plasmid. (B) Tissue fungal burden (determined by qPCR) of target organs (brains and kidneys) harvested from mice (n = 10 per arm) infected with wild-type (7 x 10^4^ spores), *R*. *oryzae* transformed with empty plasmid (5.7 x 10^4^ spores) or *R*. *oryzae* with dual attenuated expression of *FOB1* and *FOB2* (4.8 x 10^4^ spores). Data are expressed as median ± interquartile range. *y* axis value represents the lower limit of detection in the assay. *P<0.05 compared to tissues harvested from mice infected with wild-type or empty plasmid transformant. (C) Histopathological examination (H&E stain) of target organs revealed minimal inflammation and fungal elements in brains and kidneys of mice infected with RNA-i transformant, while organs collected from the empty plasmid transformant infected mice had abscesses with abundant hyphae (arrows) and neutrophil infiltration. Magnification of 400 x. (D) *In vivo* expression of *FOB1* and *FOB2* genes in brain and kidneys harvested from mice infected with wild-type, empty plasmid or RNA-i dual transformant as determined by qRT-PCR using specific primers to each of the *FOB* genes. Data are expressed as mean ± SD. * *P<*0.001 vs. wild-type or empty plasmid. Error bars represent the standard deviation of the mean from 10 mice per arm. (E) DKA mice (n = 10 per arm) were infected with *R*. *oryzae* transformed with empty plasmid (2.8 x 10^3^ spores) or *R*. *oryzae* with dual attenuated expression of *FOB1* and *FOB2* (2.4 x 10^3^ spores).

Because *FOB1* and *FOB2* are involved in iron uptake from ferrioxamine and since iron acquisition from the host is a critical virulence factor for mucormycosis [9,10,23], we hypothesized that these two genes are critical determinants of virulence especially in a mouse model of deferoxamine treatment. To test this hypothesis, we compared the virulence of *R*. *oryzae* with reduced cell surface expression of Fob1 and Fob2 proteins to wild-type or to *R*. *oryzae* transformed with empty plasmid using a deferoxamine-treated mouse model infected with a high inocula of 10^5^ spores or a lower dose of 10^3^ spores. At the higher inoculum, *R*. *oryzae* cells harboring the empty plasmid was as virulent as wild-type *R*. *oryzae* (median survival time of 3 days of the wild-type and the empty plasmid strains infected mice *P* = 0.33). In contrast, mice infected with one of two independently generated dual RNA-i transformants had attenuated virulence with > 21 day median survival time and 1/2 of the mice surviving the lethal infection (*P<*0.001). Further, at the lower inoculum of 10^3^ spores, the two RNA-i transformants were avirulent with 100% of the mice surviving the infection and appeared healthy at the termination of the experiment, while mice infected with *R*. *oryzae* transformed with the empty plasmid had 80% mortality as early as 5 days post infection (*P*<0.001) (Fig 8A). In support of these data, mice infected with the RNA-i transformant had ~ 2 log reduction in fungal burden in the brains and kidneys (primary and secondary target organs) when compared to same organs recovered from mice infected with wild-type cells or those infected with the empty plasmid transformant (*P <*0.0001) (Fig 8B). To compare the severity of infection, we conducted histopathological examination on mice organs infected with *R*. *oryzae* transformed with the empty plasmid vs. those infected with *R*. *oryzae* transformed with RNA-i plasmid targeting both *FOB1* and *FOB2*. Brains and kidneys harvested from mice infected with *R*. *oryzae* transformed with RNA-i construct had minimal inflammation, edema and minimal to no presence of fungal elements. In contrast, organs taken from mice infected with *R*. *oryzae* transformed with the empty plasmid had abundance of fungal abscesses characterized by phagocyte infiltration and substantial edema (Fig 8C).

To confirm that the RNA-i construct inhibited *FOB1* and *FOB2*, we assessed the pattern of *in vivo* expression of these genes in fungi recovered from the same mouse organs that were processed for tissue fungal burden. Relative to the fungal actin house keeping gene (*ACT1*), *FOB1* and *FOB2* were expressed in organs collected from mice infected with the wild-type, or the empty plasmid transformant. Importantly, fungal cells recovered from brain and kidneys of mice infected with *R*. *oryzae* transformed with the RNA-i construct had ~80% reduction in *FOB1* or *FOB2* gene expression (Fig 8D). These results indicate that *FOB1* and *FOB2* are expressed *in vivo* and the reduced virulence in mice infected with *R*. *oryzae* transformed with the RNA-i is due to attenuation of expression of both *FOB* genes. Finally, *R*. *oryzae* transformed with the dual construct of pFOB1/FOB2 had identical virulence to *R*. *oryzae* transformed with the empty plasmid in the diabetic ketoacidotic mice (Fig 8E). Collectively, these data show that *FOB1* and *FOB2* are required for maximal virulence of *R*. *oryzae* only in the deferoxamine-treated mice.

### Iron uptake from ferrioxamine is dependent on the reductase/permease pathway

**Fig 9:**
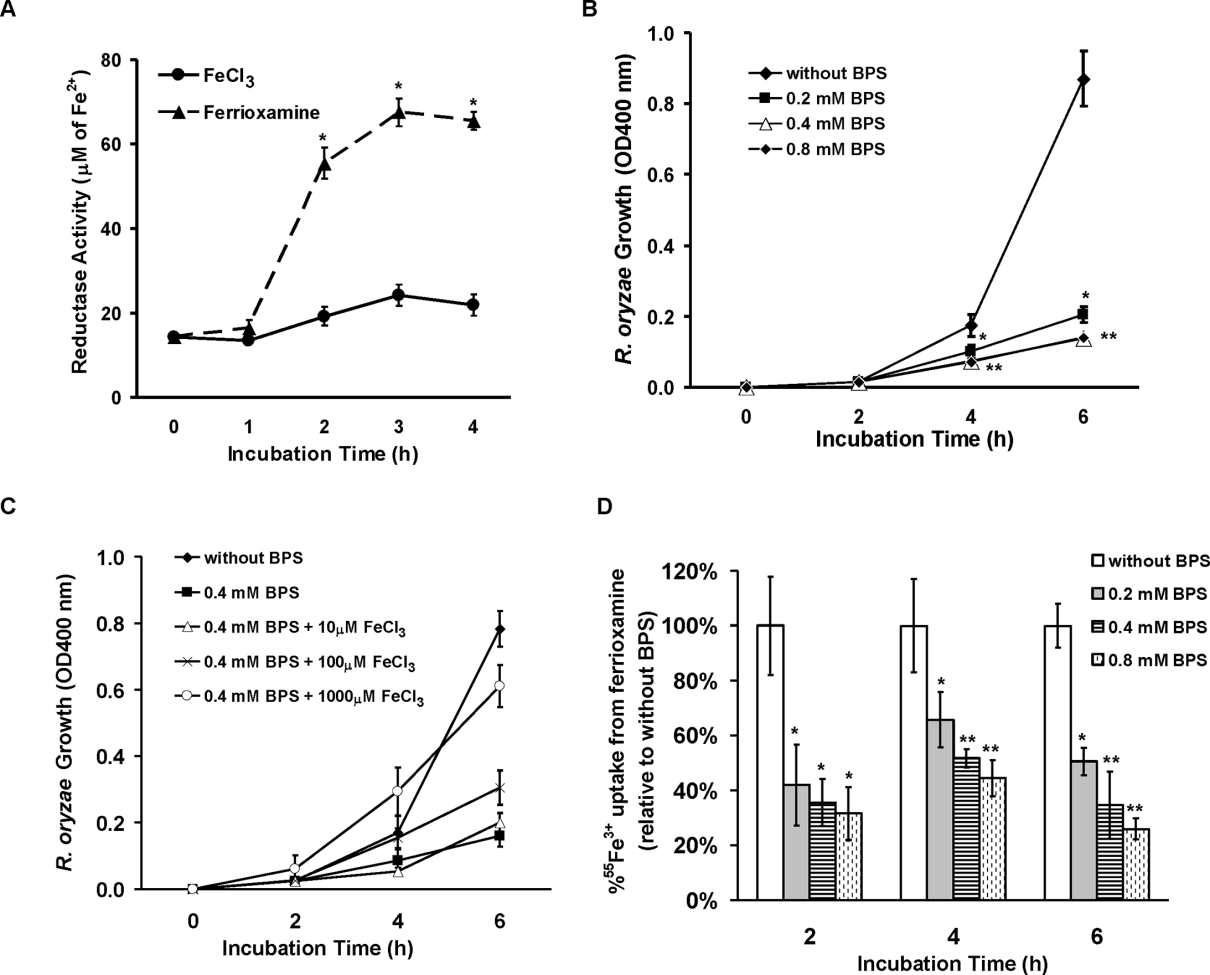
*R*. *oryzae* iron uptake from ferrioxamine is mediated by reductase activity. (A) Iron uptake from ferrioxamine is associated with increased reductase activity as compared to iron uptake from FeCl_3_ (n = 4 per each time point). *P<0.03 vs. reductase activity of FeCl_3_ at the corresponding time point. (B) The ferrous chelator BPS inhibits growth of *R*. *oryzae* on medium supplemented with ferrioxamine as a sole source of iron (n = 6 per group and per time point). *P <0.006 vs. without BPS, while **P <0.006 vs. without BPS or 0.2 mM BPS. (C) FeCl_3_ reverses growth inhibition mediated by BPS (n = 4 per group and per time point). (D) BPS inhibits ^55^Fe^3+^ uptake from ferrioxamine (n = 8 per group and per time point). * P<0.04 vs. without BPS and **P <0.003 vs. without BPS or with 0.2 mM BPS. Error bars represent the standard deviation of the mean from two independent assays.

**Fig 10:**
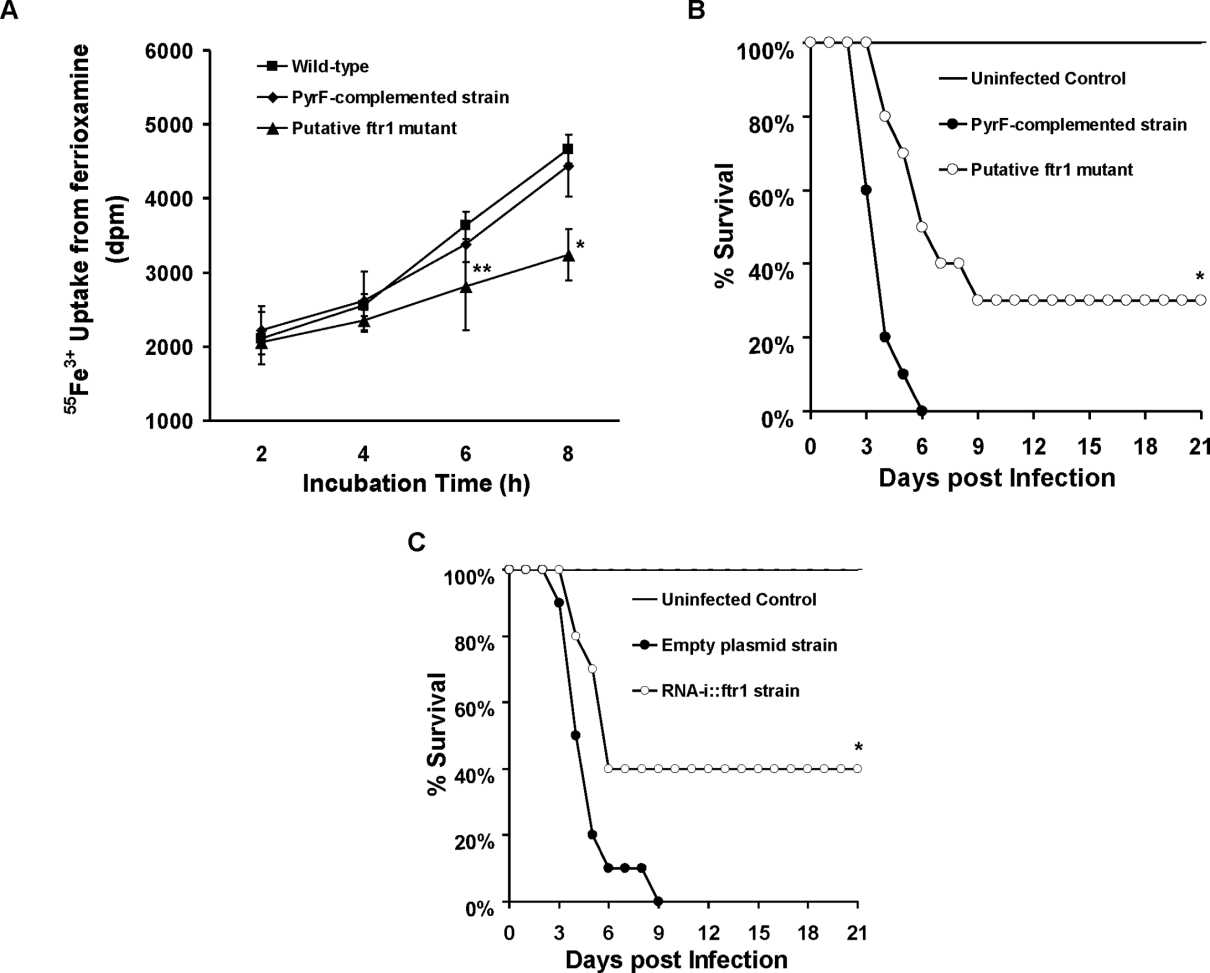
Reduction of *FTR1* expression decreases the ability of *R*. *oryzae* to take up iron from ferrioxamine *in vitro* and attenuates *R*. *oryzae* virulence in the deferoxamine-treated mice. (A) ^55^Fe^3+^ uptake from ferrioxamine by wild-type, *PyrF-*complemented, or putative *ftr1* strains (a strain with reduced copies of *FTR1* [24], n = 6 per arm from two independent experiments). *P<0.006 vs. wild-type or *PyrF-*complemented strains. **P<0.01 vs. wild-type strain. (B) Survival of deferoxamine-treated mice (n = 10 per arm) infected with *R*. *oryzae PyrF-*complemented strain (9.4 x 10^2^ spores), or *R*. *oryzae* putative *ftr1* null (6.5 x 10^2^). *P = 0.001 compared to mice infected with *R*. *oryzae PyrF*-complemented strain. (C) Survival of deferoxamine-treated mice (n = 10 per arm) infected with *R*. *oryzae* transformed with the empty plasmid (9.1 x 10^2^ spores), or *R*. *oryzae* transformed with RNA-i construct targeting *FTR1* (RNAi::*ftr1*) (9.5 x 10^2^) [24]. *P = 0.013 compared to mice infected *R*. *oryzae* transformed with empty plasmid.

Having established the importance of *FOB1* and *FOB2* in the uptake of iron from ferrioxamine *in vivo*, we wanted to investigate the mechanism by which *R*. *oryzae* obtains iron from ferrioxamine. Since one of the most common pathways of obtaining iron in fungi and *R*. *oryzae* is the reductase/permease pathway [9], we sought to investigate if this pathway plays a role in iron uptake from ferrioxamine. *R*. *oryzae* wild-type cells growing in medium containing ferrioxamine as a sole source of iron demonstrated ~ a 3 fold increase in reductase activity when compared to cells growing on medium containing iron in the form of FeCl_3_ (Fig 9A). Importantly, the extracellular, membrane-impermeable ferrous chelator bathophenanthroline disulfonate (BPS) inhibited growth of *R*. *oryzae* when ferrioxamine is used as a sole source of iron (Fig 9B). This inhibition was due to chelating ferrous since increased concentrations of FeCl_3_ reversed the inhibitory effect of BPS (Fig 9C) and the addition of BPS blocked ^55^Fe^3+^ uptake from ferrioxamine (Fig 9D). Similar results were obtained with the membrane-permeable ferrous chelator bipyridyl (S5 Fig). Since ferrioxamine chelates ferric iron, this inhibition implies the reduction of the ferric iron to ferrous prior to transportation to the fungal cell. Previously, we have shown that targeted gene disruption of *FTR1* in the multinucleated *R*. *oryzae* results in a mutant with reduced copies of the high affinity iron permease (*FTR1*) rather than a completely *ftr1* null mutant [24]. This mutant with reduced copies of *FTR1* had a retarded growth on medium containing ferrioxamine B as a sole source of iron which implies a role for Ftr1p in iron transport from ferrioxamine [24]. To determine if Ftr1p is required to transport the released ferrous to the fungal cell wall, we compared the ability of this *R*. *oryzae* strain with reduced *FTR1* copy number to wild-type or *PyrF-*complemented strain in their ability to take up ^55^Fe^3+^ from ferrioxamine. After 6 hours of incubation with radiolabeled ferrioxamine, *R*. *oryzae* with reduced *FTR1* copy number (i.e., putative *ftr1* mutant) had significant decrease in taking up ^55^Fe^3+^ compared to wild-type cells (*P<*0.01). Moreover, after 8 hours of incubation with ferrioxamine, *R*. *oryzae* with reduced *FTR1* copy number had significant reduction in ^55^Fe^3+^ uptake when compared to wild-type or *PyrF*-complemented strains (*P*<0.005) (Fig 10A).

To confirm these *in vitro* findings, we compared the virulence of two *R*. *oryzae* with attenuated expression of *FTR1* to their corresponding control strains in the deferoxamine-treated mouse model. *R*. *oryzae* strain with reduced *FTR1* copy number (Fig 10B) and *R*. *oryzae* transformed with RNA-i construct targeting *FTR1* expression [24] (Fig 10C) had attenuated virulence in the deferoxamine-treated mouse model when compared to *PyrF-*complemented strain or *R*. *oryzae* transformed with the RNA-i empty plasmid, respectively. Specifically, at day 21 post infection, 30% of mice infected with reduced *FTR1* copy number strain survived the infection vs. 0% for mice infected with the *PyrF-*complemented strain (*P =* 0.001) and 40% of mice infected with *R*. *oryzae* transformed with RNA-i construct targeting *FTR1* survived the infection vs. 0% survival for mice infected with *R*. *oryzae* transformed with the empty plasmid (*P* = 0.013). Collectively, these results clearly show that iron transport from ferrioxamine relies, at least in part, on the reductase/permease system.

### Potential role of other putative transporters in iron-uptake from ferrioxamine

**Fig 11:**
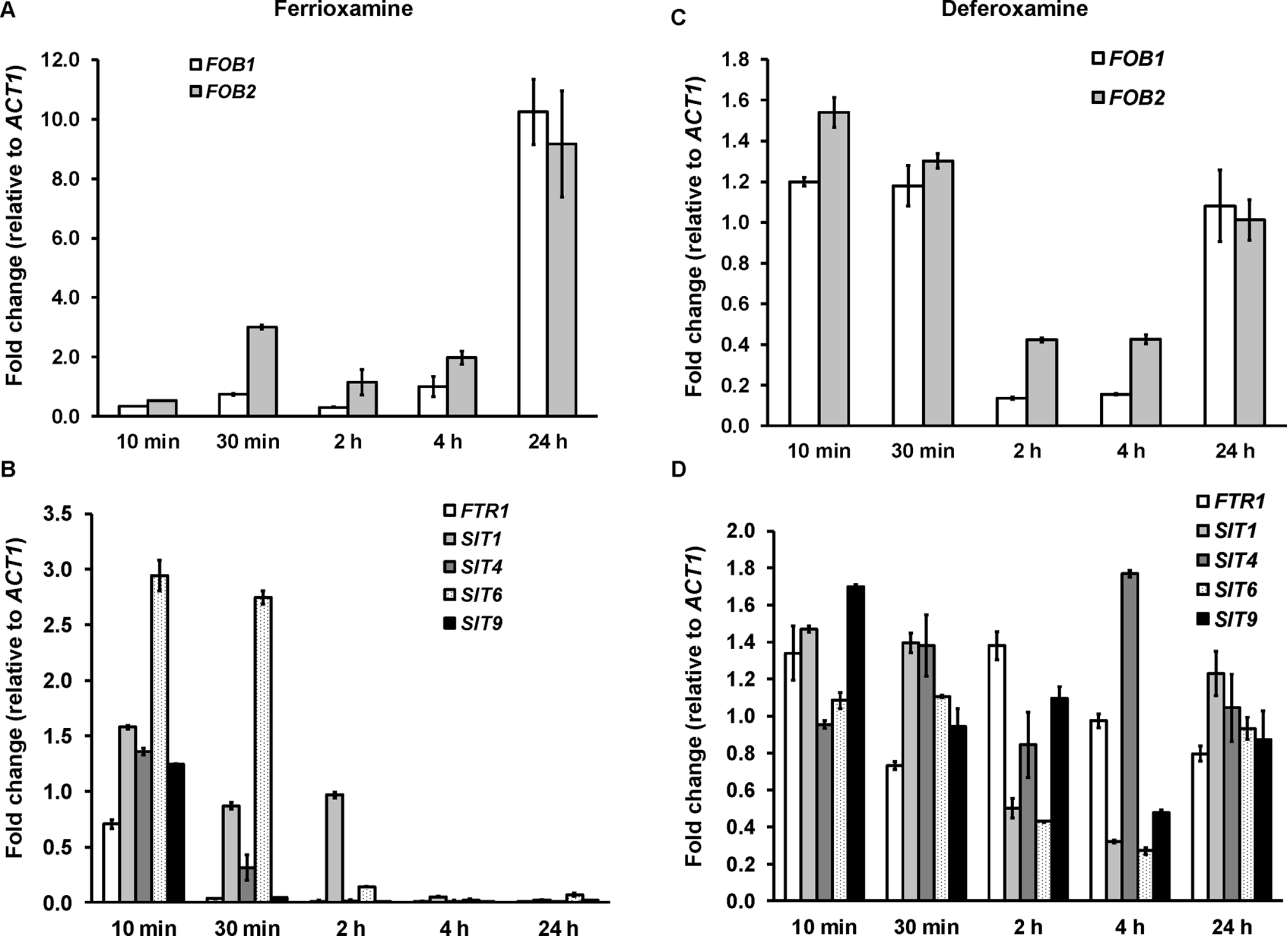
*FOB* genes are induced in ferrioxamine containing medium, while iron transporter genes such as *FTR1* and *SIT*-like genes are transiently expressed and tightly regulated. qRT-PCR was used to study the expression of *FOB1*, *FOB2*, *FTR1*, or *SIT*-like genes in media supplemented with 10 μM ferrioxamine (A, B) or deferoxamine (C, D). Only ferrioxamine strongly induced the expression of *FOB* genes (A). Iron starvation genes (*FTR1* and *SIT*-like genes) were transiently expressed in ferrioxamine-containing medium over time (B) and expressed with minimal variation in deferoxamine-containing medium (D). Error bars represent the standard deviation of the mean from two independent assays (n = 6 per group).

Because the *ftr1* mutants were not completely avirulent in deferoxamine-treated mice, we investigated the possibility of the involvement of other mechanisms in transporting iron from ferrioxamine. All identified eukaryotic siderophore transporters belong to the siderophore–iron transporters (*SIT*) family, a subfamily of the major facilitator superfamily [25,26,27,28]. We have conducted homology searches of the *S*. *cerevisiae SIT* genes [25,29,30], including *ARN1*, *ARN2*, *ARN3/SIT1*, and *ARN4* with the published *R*. *oryzae* genome data base. Although the overall amino acid identity was low (i.e. 20–23%), we identified 9 ORFs that share homology with the *S*. *cerevisiae SIT* encoding genes and therefore potentially encode Sit proteins in *R*. *oryzae* (S1 Table). We compared the pattern of expression of four of these genes (*SIT1*, *4*, *6*, and *9*) with the highest homology to *S*. *cerevisiae SIT* genes to *FOB1*, *FOB2*, and *FTR1* over time in the presence of deferoxamine or ferrioxamine as iron-poor and iron-rich media, respectively. In iron-rich ferrioxamine medium, *FOB2* expression was induced as early as 30 min and reached a maximum expression of 9 fold increase by 24 hrs. Interestingly, *FOB1* expression was significantly increased only after 24 h incubation (Fig 11A). This enhancement of *FOB* gene expression was accompanied by gradual reduction in iron starvation genes (i.e. *FTR1* and *SIT*) that reached >99% decrease after 24 h (*FTR1* and *SIT9* had almost complete reduction in expression after 30 min of incubation) when compared to 10 min data set (Fig 11B). In contrast and as expected, none of the *FOB* gene expression was enhanced in the presence of deferoxamine (Fig 11C). Finally, *FTR1* and putative *SIT* gene level of expression did not considerably change in deferoxamine containing medium over time (level of expression fluctuated between 0.3–1.4 fold relative to *ACT1*) (Fig 11D). These results confirm the induction of *FOB* genes in response to ferrioxamine and that this induction changes the conditions of the fungal cell from iron-deplete to iron-replete conditions as shown by the reduction in the iron-starvation gene expression. Further, the similar pattern of *SIT* genes expression to the *FTR1* expression in ferrioxamine containing medium suggests that these genes might play a role in iron uptake from this siderophore.

## Discussion

The unique predisposition of dialysis patients receiving deferoxamine for treating iron overload to mucormycosis has been clinically known for decades [13,15,16]. Previous data demonstrated that *Rhizopus* species can utilize iron from ferrioxamine as a xenosiderophore to obtain iron from the host [17,31,32]. This process was found to be energy dependent and possibly doesn’t require internalization of the siderophore [18]. In this study, we have identified two ORFs that encode Fob1 and Fob2 proteins which are required for iron uptake from ferrioxamine. We introduce evidence that these proteins are receptors to which ferrioxamine binds to during the process of obtaining iron by *R*. *oryzae*. First, Fob proteins are mainly induced by ferrioxamine and detected in the supernatants of regenerating *R*. *oryzae* protoplasts before they are covalently incorporated into the nascent cell surface [33]. Second, these two proteins were found to be expressed on the cell surface of *R*. *oryzae*, a criterion required for cell receptors. Third, whole cell extracts from *R*. *oryzae* grown in the presence, but not the absence, of ferrioxamine bound to radiolabeled ferrioxamine under non-denaturing conditions. Fourth, Fob2 protein isolated from whole cell extracts using anti-Fob2 antibodies also bound to radiolabeled ferrioxamine. Finally, a mutant of *R*. *oryzae* with attenuated *FOB1* and *FOB2* expression bound much less, and had retardation in, iron uptake from radiolabeled ferrioxamine versus the wild-type strain. Collectively, these features indicate that Fob1 and Fob2 proteins are ferrioxamine induced, surface exposed, and directly interact with the siderophore.

The identified Fob proteins were found to belong to the CBS domain family. CBS domain is a conserved protein sequence region, which is found in all kingdoms of life [34]. It was first described in Cystathionine β Synthase, hence the name CBS. Other proteins belonging to this family include inosine monophosphate dehydrogenase, AMP kinase, and chloride channels. Therefore, CBS-containing proteins functions are very diverse and could range from affecting metabolism, multimerization and sorting of proteins, channel gating, to ligand binding. Based on their function, CBS domain proteins can be present in the cytoplasm or associated with the cell membrane [34]. For example, despite the lack of classical features of secreted proteins such as N-terminal signal peptide sequence, CBS domain proteins were reported to be associated with cell membrane in eukaryotic cells functioning as CLC chloride channels with outer membrane sequence, transmembrane domain, and a cytoplasmic tail [35]. Concordant with this report is our finding that Fob1p and Fob2p are predicted to be surface associated by using the MEMSAT program (S1 Fig). In most cases, however, CBS-domain proteins bind to ligands related to adenosine, including S-adenosylmethionine (SAM), AMP, ADP [36], as well as recently reported DNA and RNA fragments [37,38]. They also have been reported to bind to metallic ions such as Mg^2+^ [39]. To our knowledge, we show for the first time that a ferrioxamine receptor belongs to the CBS domain family of proteins. Given the diverse function of these proteins and the previous report of binding metallic ions, it is possible that Fob1 and Fob2 proteins on *R*. *oryzae* cell surface bind ferrioxamine via Fe^3+^. This assumption is supported by our results that only ferrioxamine, but not deferoxamine, induces the expression of *FOB1* and *FOB2* and by the fact that deferoxamine was not able to competitively inhibit the uptake of radiolabeled ferrioxamine by *R*. *oryzae* Importantly, ferric-rich siderophores are known to induce a conformational change of the secondary structure of their receptors and likely explain the ability of ferrioxamine and inability of deferoxamine to induce and bind to Fob proteins [40,41].

An apparent discrepancy in our results is the actual size of the Fob2p of ~40 kDa as indicated by the predicted sequence, the size of the rFob2p and recognized band from whole cell extracts vs. the originally isolated 70 kDa band. We initially assumed that the presence of multiple possible *N*- and *O*-glycosylation sites might account for this increase in size of the protein due to post-translational modification. However, the isolation of Fob proteins from whole *R*. *oryzae* extracts at the predicted size of 40 kDa argues against a post-translational modification of the protein. A more likely explanation is the possible oligomerization of the protein when the sample is present in concentrated form mixed with SDS. First, the 70 kDa band was detected in concentrated supernatants from regenerating *R*. *oryzae* protoplasts in SDS-PAGE. Second, when we immunoprecipitated whole cell extracts from wild-type cells, the majority of the sample remained bound to the antiFob2p antibody-coated beads and that sample contained Fob proteins at the ~40 and ~70 kDa bands when separated on SDS-PAGE. It is prudent to mention that cell membrane proteins are notorious for oligomerization even in denaturing conditions when mixed with SDS [42,43]. In fact, a study found that SDS enhances the dimerization of β-amyloid proteins from human cortical tissues and the level of this unnatural dimerization increases with the increased concentration of SDS in the sample [44]. Alternatively, CBS domain proteins were reported to oligomerize (e.g. *Streptococcus pyogenes* IMPDH protein [34]), therefore it is possible that Fob proteins are present as homo- or heterodimers. Our sequence data which identified Fob2p in two independent samples detected at ~70 kDa without the identification of Fob1p suggest that Fob2p is the major form expressed. However, it is possible that Fob1p is expressed in low quantities and is required for the receptor activity by forming a heterodimer with Fob1p. Investigations into the possibility of homodimer vs. heterodimer formation of Fob2p and Fob1p or the possibility of induction of unnatural oligomerization during sample processing are currently underway.

Deferoxamine is a siderophore belonging to the hydroxamate family that is mainly isolated from bacteria including Gram-positives (e.g., *Streptomyces* and *Nocardia*) as well as Gram negatives Enterobacteriaceae [45,46,47]. Up to now, deferoxamine has not been isolated from fungi but many studies indicated the utilization of this siderophore by fungi in obtaining iron including *Mucorales*, *Aspergillus*, *Cryptococcus* [17] and *S*. *cerevisiae* [25,27,48]. In addition to utilizing deferoxamine as xenosiderophores, *Mucorales* are known for secreting their own siderophore, rhizoferrin [22,49]. However, rhizoferrin belongs to the carboxylate family which is structurally distinct from the hydroxamate siderophores [22,50]. Interestingly, rhizoferrin (with or without iron) did not induce the expression of *FOB1* or *FOB2* indicating that these genes encode receptors specific to iron uptake from ferrioxamine. However, the ability of *Mucorales* to utilize iron from structurally distinct siderophores points to the critical role iron plays in the survival of this group of fungi.

Our *in vitro* data with down regulation of a single *FOB* gene demonstrated a modest effect on germination, growth and iron uptake from medium supplemented with ferrioxamine. These results can be explained by the fact that the untargeted gene was overexpressed and had a compensatory effect. This compensatory mechanism by related genes is well documented when other genes of the family are nullified [51]. However, this compensatory gene expression effect did not translate into full restoration of cell surface protein to the level of *R*. *oryzae* transformed with empty plasmid and grown in ferrioxamine containing medium (Fig 4D). The reduction of fluorescence in a single inhibition strain could be due to the possibility that the enhancement of non-targeted gene expression did not rise to the level that would entirely compensate for inhibition of the other gene. It is also possible that the gene expression of the non-targeted gene does not localize entirely to the cell surface.

Our strategy of targeting both genes with a single construct successfully drastically attenuated the expression of *FOB1* and *FOB2*, resulting in a significant retardation in germination, growth and iron uptake from medium supplemented with ferrioxamine as a sole source of iron. However, the inhibition of growth was not complete. Further, the inhibition of iron uptake from ferrioxamine by *R*. *oryzae* with abrogated *FOB1*/*FOB2* expression was marginally reduced at later time points (Fig 7C, compare 4 h with earlier time points). These two phenomena can be explained by the fact that old or heat-damaged ferrioxamine (due to incubation at 37^o^C) is unstable and undergoes an autoreduction step to release ferrous iron [52], that is taken up by the fungus. This assumption is corroborated by the yielding of a pink-colored complex in the presence of ferrozine in our experiment. It is also probable that secondary mechanisms of obtaining iron from ferrioxamine which are independent from *FOB1/FOB2* are operative. Our results with pattern of expression of *SIT*-like genes in medium supplemented with ferrioxamine or deferoxamine over time supports the model of *SIT-*like genes being involved in iron uptake from ferrioxamine. Specifically and similar to *FTR1* gene, *SIT-*like genes were transiently expressed as early as 10 min then the expression faded over time which indicates change of the conditions of the fungal cell from iron-deplete to iron-replete conditions. This transient expression and tight regulation is critical for the organism to avoid the toxic carnage of excess iron [53,54].

Importantly, a *R*. *oryzae* mutant with dual attenuation of *FOB1/FOB2* expression had severe virulence retardation in the deferoxamine-treated mice vs. mice infected with wild-type or empty plasmid strains with 50% of the mice infected with *R*. *oryzae* transformed with RNA-i targeting *FOB1/FOB2* surviving the infection and looking healthy. It is imperative to point out that this difference in virulence was not detected in the DKA mouse model which is consistent with the fact that Fob1 and Fob2 are proteins induced by ferrioxamine. Fob1 and Fob2 are likely dispensable in the DKA model since Fe^3+^ is released from transferrin by the action of acidosis and hyperglycemia [9,11] and transported into the fungal cell via the Ftr1p [24].

**Fig 12:**
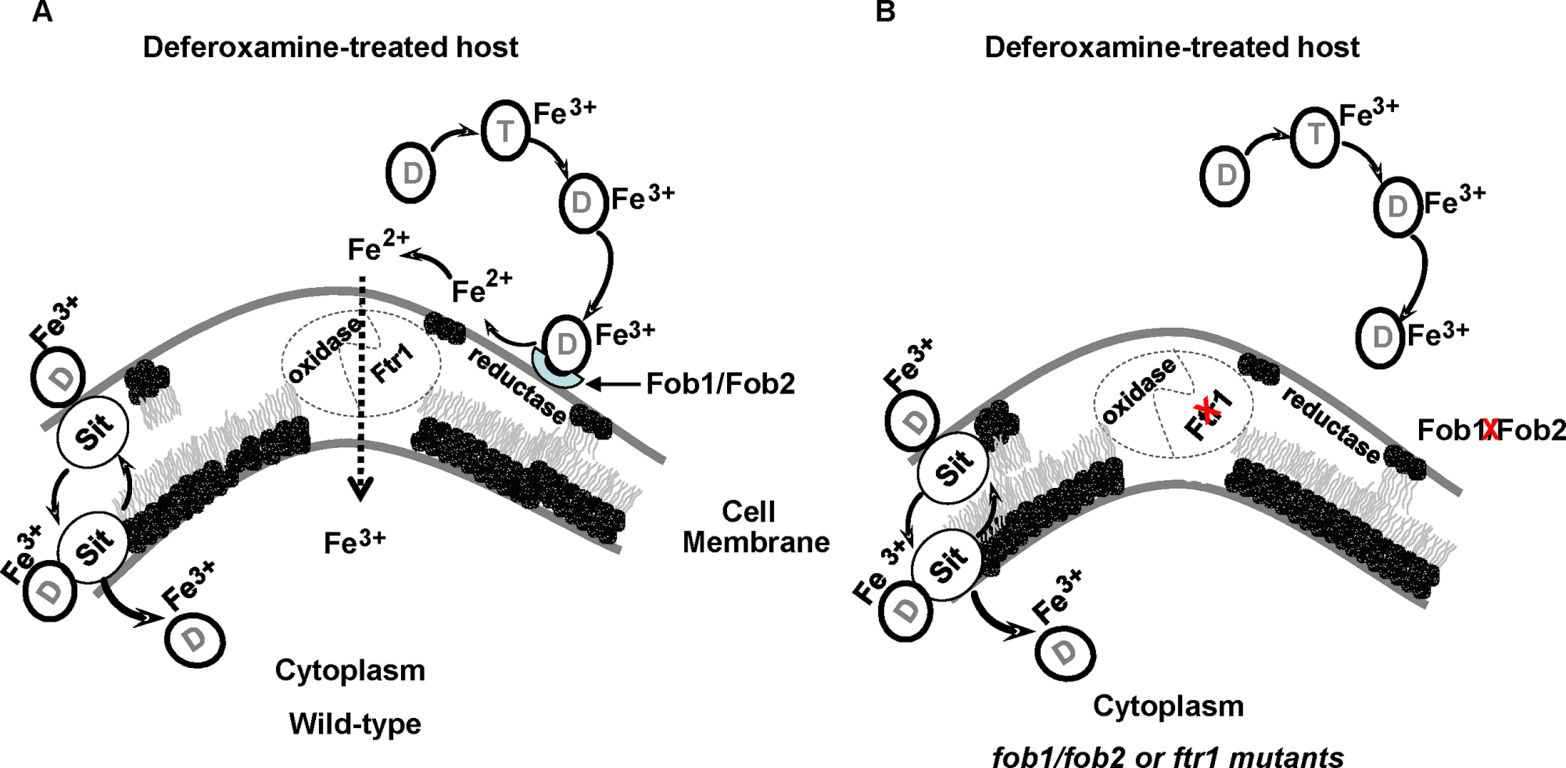
Proposed mechanism of iron uptake by *R*. *oryzae* from ferrioxamine. Deferoxamine (D) directly chelates iron from transferrin (T), resulting in ferrioxamine which binds to Fob1 and/or Fob2 proteins on the cell surface. *R*. *oryzae* then liberates Fe^2+^ iron by the actions of cell surface reductases then transported into the cell by oxidase/Ftr1 complex (A). In the absence of Fob1/Fob2 or Ftr1 proteins, the ability of *R*. *oryzae* to obtain iron from ferrioxamine is compromised and the virulence is abrogated in deferoxamine-treated mouse model (B). Sit-mediated shuttle mechanism of ferrioxamine transportation probably acts as a secondary mechanism of iron uptake from ferrioxamine.

In microorganisms, the mechanism of iron uptake from ferrioxamine involves either the uptake of the entire siderophore complex, reduction of the Fe^3+^ to Fe^2+^ to release it from the siderophore followed by uptake of iron via oxidase/permease system, or both. For example, in *S*. *cerevisiae* it has been reported that both mechanisms are operative [30,55]. A previous study demonstrated that iron uptake from ferrioxamine by *R*. *oryzae* is likely energy dependent, require a reductive step and doesn’t involve the uptake of the ferrioxamine complex [18]. In contrast, by using a fluorescent derivative of deferoxamine, a recent study showed that the entire siderophore was taken up by the *R*. *oryzae* cells [56]. Our results mainly support the model by which *R*. *oryzae* obtains iron from ferrioxamine via the reductase/permease system. First, the reductase activity of *R*. *oryzae* when incubated with ferrioxamine far exceeds the reductase activity when fungal cells were incubated with FeCl_3_. Second, the addition of the ferrous chelator BPS inhibited growth of *R*. *oryzae* in medium with ferrioxamine as a sole source of iron and this inhibition was due to attenuation of iron uptake from ferrioxamine. Finally, we previously reported that a *R*. *oryzae* mutant with reduced copy number of the high affinity iron permease (*FTR1*) had reduced growth on medium supplemented with ferrioxamine [24]. In this study we show that this growth inhibition is likely due to the diminished ability of this mutant to take iron from ferrioxamine. More significantly, we demonstrated reduced virulence in the deferoxamine-treated mouse model of mucormycosis, even though the *R*. *oryzae* mutant with reduced *FTR1* copy number or inhibited expression of *FTR1* do not have full abrogation of the function of the permease activity [24]. However, a minor mechanism reliant on internalization of the entire siderophore cannot be completely ruled out because the effect of BPS *in vitro* and the attenuated virulence with *ftr1* mutants were not complete. It is possible that the reductase/permease pathway represent a major uptake pathway for iron acquisition from ferrioxamine, while the uptake of the siderophore by shuttle mechanism[56] represents a secondary mechanism. This secondary mechanism of iron uptake from ferrioxamine is supported by the pattern of transient and tightly regulated *SIT-*like gene expression in ferrioxamine containing medium (Fig 11). Also the assumption that ferrioxamine is taken by a Sit-like shuttle transporter is a secondary mechanism is enforced by the fact that the patchy distribution of fluorescent ferrioxamine was observed only at the later time point of 24 hours of incubation and not earlier [56]. Consequently, we propose that *R*. *oryzae* overexpresses Fob1 and Fob2 in response to ferrioxamine, which accumulates in a host being treated with deferoxamine to eliminate iron overload toxicity. These receptors bind ferrioxamine and through fungal cell surface reductase activity, Fe^3+^ is released from the siderophore as Fe^2+^ prior to being transported across the fungal cell membrane by the oxidase/permease complex (i.e., Fet3/Ftr1) to support fungal growth and virulence (Fig 12A). In the absence of Fob1/Fob2 or the Ftr1p, this process is compromised and the fungal virulence is reduced (Fig 12B). Also in this model, transport of the entire ferrioxamine siderophore by Sit-like transporter is likely to be operative as a secondary pathway.

In summary, we have identified Fob1 and Fob2 proteins as ferrioxamine receptors in *R*. *oryzae*. These cell surface proteins are induced by ferrioxamine and are required for mucormycosis pathogenesis in the deferoxamine-treated mouse model. The mechanism of iron uptake from ferrioxamine is reliant on binding of the iron-rich siderophore to its receptor, followed by transport of iron to the fungal cell via the reductase/permease system without internalization of the siderophore-iron complex. These two receptors appear to be conserved in at least another five *Mucorales* and can be the subject of future novel therapy to maintain the use of deferoxamine for treating iron-overload.

## Materials and Methods

### Organisms and culture conditions

All organisms used in this study are listed in S2 Table. *R*. *oryzae* 99-880 is a clinical strain isolated from a patient with a brain abscess and obtained from the Fungus Testing Laboratory of

University of Texas Health Science Center at San Antonio. The organism was routinely grown on potato dextrose agar (PDA) plates (BD) for 4-5 days at 37°C. *R*. *oryzae* M16 is a *pyrF* null mutant that is derived from *R*. *oryzae* 99-880 and is unable to synthesize its own uracil [57], was grown on YPD medium (MP Biomedicals) supplemented with 100 μg/ml uracil. *R*. *oryzae PyrF-*complemented strain, *R*. *oryzae* with reduced *FTR1* copy number, and *R*. *oryzae* transformed with RNA-i targeting *FTR1* expression were all derived from strain 99-880 and M16 and previously described in detail [24]. In RNA-i experiments, a chemically-defined synthetic medium containing yeast nitrogen base (YNB) supplemented with complete supplemental mixture without uracil (CSM-URA) (MP Biomedicals) (i.e., YNB+CSM-URA) (formulation/liter, 17 g yeast nitrogen base without amino acids (YNB) (BD), 200 g dextrose, and 7.7 g complete supplemental mixture minus uracil (CSM-URA)) was used. To count and isolate single colonies all media above were supplemented with 0.1% Triton X-100 (Sigma). Spores were collected in endotoxin free PBS containing 0.01% Tween 80, washed twice with PBS, and counted with a hemacytometer for inoculum preparation.

### Isolation of ferrioxamine putative receptors

To identify the *R*. *oryzae* cell surface protein(s) that interacts with ferrioxamine, freshly harvested *R*. *oryzae* 99-880 spores (2.5x10^8^) were added to 50 ml YPD and pregerminated with shaking at 37°C. After 4 hours, the spores were pelleted by centrifugation and washed twice with 0.5 M sorbitol buffer, and suspended in 50 ml protoplasting solution which consists of 10 mM sodium phosphate, pH 6.4, 0.5 M sorbitol, 250 μg/ml lysing enzyme (Sigma), 150 μg/ml chitinase (Sigma) and 150 μg/ml chitosanase (US Biologicals). The suspension was incubated for ~ 2 h at 30°C with gentle shaking (100 rpm) until majority of the spores (> 90%) formed protoplasts as determined by light microscopy examination and their sensitivity to water. Protoplasts were then collected by centrifugation at low speed (100 rpm), washed twice with 0.5 M sorbitol, and then resuspended in 10 ml YNB with amino acids containing 0.5 M sorbitol in the presence or absence of 10 μM ferrioxamine (Sigma). The protoplasts were allowed to regenerate for 2 h by incubating at 37°C with gentle shaking at 100 rpm. During the early stages of this regeneration process, many surface protein precursors are shed into the extracellular medium but not yet covalently incorporated into the nascent cell surface, thereby enabling their easy isolation and identification [33]. Thus, supernatants containing cell wall materials from regenerated protoplasts were isolated from fungal cells by centrifugation at 1000 rpm, followed by filtration through a 0.25 μm pore-size membrane after the addition of Halt Protease Inhibitor Cocktails (Thermo Scientific). The filtrate was then concentrated using iConcentrator tube with molecular weight cutoff = 9.0 kDa (Thermo Scientific) and protein concentration was measured with the Bradford method (Bio-Rad). Proteins from supernatant of regenerated protoplasts in the presence or absence of ferrioxamine were separated on 10% SDS-polyacrylamide gel (Bio-Rad) and stained with Coomassie dye R-250 (Pierce). To investigate if the proteins from supernatant of regenerated protoplasts bind to ferrioxamine B, 25 μg of protein extract was mixed with ^55^Fe-labeled ferrioxamine (for preparation, see below), and incubated for 1 h at room temperature. The mixture was then mixed with native protein sample buffer and separated by running a native gel (Bio-Rad) in the absence of SDS and exposed to X-ray film. The candidate band from SDS-PAGE was cut and microsequenced using MALDITOF MS/MS (UCLA Molecular Instrumentation Center).

### Preparation of radiolabeled ferrioxamine

^55^Fe^3+^ labeled ferrioxamine was prepared as previously described [58]. Briefly, ^55^FeCl_3_ (3 μl of 3.5 Ci/mmol, Perkin Elmer) was mixed with iron-free deferoxamine B (Sigma) in a molar ratio of 0.9:1 in a volume of 100 μl, and incubated for 2 h at room temperature. Free iron was then removed by adding sodium phosphate (pH 7.4) followed by centrifugation at 20,000 g for 2 min. Supernatant was diluted to 3.0 ml with ultra-pure water with a resistivity of 18.2 megohm-cm (Barnstead International, Dubuque, Iowa) and treated with 150 mg Chelex 100 resin (Bio-Rad) twice to get rid of any residual free iron.

### *FOB1*, *FOB2*, and *SIT* transporter expression *in vitro*

To quantify gene expression of putative ferrioxamine receptors and other *SIT* genes of *R*. *oryzae in vitro*, total RNA was isolated from mycelia that have been grown in YNB with amino acids containing 10 μM of siderophore (ferrioxamine, deferoxamine, rhizoferrin with or without ferric iron [EMC Microcollections GmbH])) or varying concentrations of FeCl_3_ overnight with shaking at 37°C. The RNA was isolated using RNeasy Plant Mini Kit (Qiagen) after grinding the mycelia in liquid nitrogen. cDNA was synthesized from 2 μg of total RNA from each sample after treating with Turbo DNA-free DNase I (Life Technologies) to remove any contaminating DNA. After removing DNase I with DNase inactivation Reagent (Life Technologies), cDNA was synthesized using RETROscript kit (Life technologies). qPCR was carried out using the Power SYBR Green method in the StepOne Real-Time PCR System (Life Technologies) with a thermal-cycling program as follows: initial denaturing step for 10 min at 95°C, followed by 40 cycles of denaturing at 95°C for 15 s, and annealing/elongation at 60°C for 1 min. *R*. *oryzae* actin gene (*ACT1*) was used as a reference control, putative ferrioxamine receptor gene-specific primers are listed in S3 Table. The comparative Ct method was used for analysis [59].

### Attenuation of *FOB1* and *FOB2* expression by RNAi

RNA-i was used to silence *FOB1* or *FOB2* expression individually or collectively. For individual silencing, two reverse-complemented fragments (~450 bp) were PCR-amplified and cloned into plasmid pRNAi-pdc-intron in an opposite direction and separated by a 100 bp-intron sequence, hence the total insert is ~1 kb [24]. Two constructs, termed pRNAi-FOB1 and pRNAi-FOB2, targeting *FOB1* and *FOB2* expression were respectively generated. To silence both genes at the same time, the 1 kb insert from pRNAi-FOB1 was cut and re-cloned downstream of pRNAi-FOB2, separated by a 50-bp interval of random sequence to yield pFOB1/2. All constructs were transformed into *R*. *oryzae* M16 (*pyrF* null mutant) using a biolistic delivery system (Bio-Rad) as previously described [60] and transformants were selected on YNB+CSM-URA plates supplemented with 0.1% triton. Primers used for this work are listed in S3 Table.

### Growth rate determination

To compare growth rate among *R*. *oryzae* isolates on medium with ferrioxamine as a sole source of iron, 10^5^ spores of each strain were plated in the middle of the YNB+CSM-URA agar plates which have been treated with 1 mM of ascorbic acid (Sigma) and 1 mM ferrozine (Sigma) to chelate iron and then supplemented with 10 μM ferrioxamine. The plates were incubated at 37°C and the diameter of the colony was calculated after 24 or 48 h.

To determine the effect of the ferrous chelators BPS or bipyridyl on the growth of *R*. *oryzae* in the presence of ferrioxamine, *R*. *oryzae* wild-type spores (5x10^5^/ml) were added to YNB+CSM-URA medium supplemented with 10 μM ferrioxamine and one of the two ferrous chelators (0.2–0.8 mM of BPS or 0.2–0.4 mM bipyridyl). In some experiments, FeCl_3_ was added in increased concentrations (10–1000 μM) to investigate if iron can reverse the effect of the ferrous chelator. Cultures were incubated at 37°C with shaking. At selected time intervals, 1 ml sample was taken from the culture and the optical density measured at 400 nm [17].

### Germination studies

Spores from *R*. *oryzae* wild-type, transformants transformed with empty plasmid or RNA-i constructs targeting *FOB1*, *FOB2*, or *FOB1/FOB2* were harvested from their plates as above and inoculated into YNB+CSM-URA medium supplemented with 10 μM ferrioxamine as a sole source of iron at 5 x 10^6^ spores/ml. The cultures were incubated at 37°C with shaking (200 rpm). At selected time periods, a 10 μl sample was collected from each culture, and examined with a phase contrast microscope to determine if there is any effect of *FOB* gene silencing on germination. Pictures were taken with a Nikon E5400 camera.

### Iron uptake

To determine the ability of different strains of *R*. *oryzae* to take up iron from ferrioxamine, we used our previously described methods [24]. Briefly, *R*. *oryzae* spores (5 x 10^6^/ml) were pre-germinated for 3 h in YNB medium supplemented with 1 mM ferrozine and 1 mM ascorbic acid at 37°C with shaking. Cells were harvested by centrifugation, washed twice with assay buffer (YNB + 10 mM 4-morpholinepropanesulfonic acid + 1 mM ferrozine), and resuspended in 7 ml of assay buffer supplemented with 10 μM of ^55^Fe-labeled ferrioxamine at a concentration of 5 x 10^6^ spores/ml, and incubated at 37°C with shaking. In some experiments, to determine if iron uptake from ferrioxamine required the reduction of Fe^3+^ to Fe^2+^, the ferrous chelator BPS (0.2–0.8 mM) or bipyridyl (0.2–0.4 mM) were added to the assay buffer. At different time points, 1 ml of sample was collected by Whatman GF/C glass filters (GE Healthcare) through a vacuum filtration manifold (Millipore) and washed twice with cold water. Cell associated ^55^Fe was counted in a liquid scintillation counter (Packard Instrument, Downers Grove, IL).

For competition studies using cold deferoxamine or ferrioxamine, ^55^Fe^3+^ uptake studies were conducted as above while including increasing concentrations of cold deferoxamine or ferrioxamine. Iron uptake was determined after 2 h incubation period and the results expressed as % inhibition relative to samples run without the inclusion of cold siderophores.

### Reductase activity

To determine the reductase activity of *R*. *oryzae* wild-type in the presence of ferrioxamine or FeCl_3_, we used the method of Dancis *et al*. [61]. Briefly 5x10^7^ of *R*. *oryzae* spores were cultured in 10 ml of YNB+CSM-URA medium supplemented with 100 μM FeCl_3_ or 10 μM ferrioxamine. The culture was incubated at 37°C with shaking. At time intervals, 1 ml of the culture was collected, centrifuged at 3000 rpm and the spores washed twice with cold distilled water. The spores were suspended in 1 ml of assay buffer (0.05 M sodium citrate (pH 6.5) supplemented with 5% glucose (w/v)). The culture was incubated at 30°C for 15 min before adding BPS and FeCl_3_ to a final concentration of 1 mM for both compounds. The mixture is vortexed and then incubated for additional 20 min, after which spores were removed by centrifugation and the optical density of the supernatant determined at 535 nm using a blank processed similarly but without any added spores. The amount of generated Fe^2+^ was estimated with a standard curve constructed from solutions of known Fe^2+^ concentrations using FeSO_4_.

### Fob2 protein purification and antibody production

To determine the cell surface localization of the putative ferrioxamine receptors, we sought to raise polyclonal antibodies against one of the two proteins (i.e., Fob1p or Fob2p). Since Fob1 and Fob2 predicted proteins are ~ 80% identical at the amino acid level, we chose to express *FOB2* (RO3G_11000). To express *FOB2*, we first PCR-amplified *R*. *oryzae FOB2* gene (S3 Table), the PCR product was gel-purified and digested with *BamH*I/*Pst*I before cloning into *E*. *coli* expression vector pQE32 (Qiagen) to yield pQE32/*FOB2*. This resulted in a construct with 6xhis tag on the N-terminus of Fob2 protein. Plasmid pQE32/*FOB2* was transformed into *E*. *coli* XL-10 gold (Agilent Technologies) and transformants isolated on Luria*-*Bertani (LB) agar plates supplemented with ampicillin (100 μg/ml). Gene cloning was confirmed by DNA sequencing. To purify Fob2 protein, a single colony from the transformed *E*. *coli* was grown overnight. On the second day, the culture was diluted 200 times in fresh medium containing ampicillin, and incubated with shaking at 37°C until reaching an OD = 0.6. At this stage, 1 mM IPTG was added to the culture which was then incubated at 37°C with shaking for additional four hours. The bacterial cells were then harvested by centrifugation, and 6xhis tagged Fob2p was purified to >90 homogeneity with HisPur Cobalt Purification Kit according to the instructional manual (Pierce). The purified recombinant protein was extensively dialyzed in phosphate buffered saline (PBS) (Mediatech, VA), and the purity and size confirmed by SDS-PAGE analysis followed by MALDI-TOF–mass spectrometry/mass spectrometry analysis.

To generate anti-Fob2p antibodies, normal BALB/c mice were immunized by subcutaneous injection of 20 μg recombinant Fob2p mixed in equal amounts with complete freund’s adjuvant (CFA, Sigma-Aldrich). The mice were boosted with a subcutaneous administration of a similar dose of the protein mixed with incomplete freund’s adjuvant (IFA) three weeks following the initial immunization. Mice vaccinated with the diluent (i.e., PBS) mixed with CFA/IFA without rFob2p using the same regimen served as control mice. Twelve days after the boost, blood samples were collected from mice, and anti-Fob2p Ab titers were determined by using ELISA plates coated with Fob2p as we previously described [62]. The ELISA titer was taken as the reciprocal of the last serum dilution that gave a positive OD reading (i.e., more than the mean OD of negative control samples plus 2 standard deviations).

### Localization of Fob1 and Fob2 to the cell surface of *R*. *oryzae*

To prove Fob proteins are *R*. *oryzae *cell surface proteins, freshly harvested spores grown on YNB+CSM-URA were pregerminted for 3–4 hours in the presence or absence of 10 μM deferoxamine. The pregerminated spores were incubated with the anti-Fob2p IgG at 1:100 for 1 hour on ice after a blocking step using 1.5% goat serum in PBS for 1 hour. Cells were washed 3 times with Tris-buffered saline (TBS; 0.01 M Tris HCl [pH 7.4], 0.15 M NaCl) containing 0.05% Tween 20, then counterstained with alexa 488-conjugated anti-mouse secondary at 1:100 for 1 hour on ice. The stained cells were imaged with Leica confocal microscope using an excitation wavelength of 488 nm.

To quantify the expression of Fob proteins on *R*. *oryzae* cell surface, the pregerminated spores were stained as above, then 1 ml samples were analyzed using a FACSCalibur (Becton Dickinson) instrument equipped with an argon laser emitting at 488 nm. Fluorescence emission was read with a 515/40 bandpass filter. Fluorescence data were collected with logarithmic amplifiers. The mean fluorescence intensities of 10^4^ events were calculated using CELLQUEST software [20,63].

### Whole cell extraction, secreted proteins, western blotting and ferrioxamine binding studies

*R*. *oryzae* (5 x 10^7^ cells) were grown overnight in YNB+CSM-URA with or without 10 μM ferrioxamine. Mycelia were collected by filtration, washed briefly with PBS, and then ground thoroughly in liquid nitrogen using pestle and mortar for 3 min. The ground powder was immediately transferred to microfuge tube containing 500 μl extraction buffer which consisted of 50 mM Tris-HCl, pH 7.5, 150 mM NaCl, 10 mM MgCl_2_. The extraction buffer was supplemented with 1X Halt Protease Inhibitor Cocktails (Thermo Scientific). The sample was vortexed vigorously for 1 min, then centrifuged for 5 min at 21000 g at 4°C. The supernatant was transferred to a new tube and the protein concentration determined using Bradford method.

For detecting protein secretion, supernatants from *R*. *oryzae* cells growing in YNB+CSM-URA with or without 10 μM ferrioxamine for overnight, were collected after filtration of the hyphal mate using 0.22 μM membrane units (Millipore). The cell-free supernatants were concentrated by 200 x and the total proteins measured as above.

For Western blotting, 5 μg of each sample was used to separate proteins on an SDS-PAGE. Separated proteins were transferred to PVDF membranes (GE Water & Process Technologies), and treated with Western blocking reagent (Roche) for 1 h at room temperature. PVDF membranes were then probed with mouse anti-Fob2p antibodies (1:100 diluted). After washing 3 times with TBS-T buffer (Tris-buffered saline + 0.05% Tween 20), the membranes were incubated with HRP-conjugated sheep anti-mouse IgG (1:10,000 dilution) (Sigma-Aldrich). Fob bands were visualized by adding the HRP substrate (SuperSignal West Dura Extended Duration Substrate, Thermo Scientific), and the chemiluminescent signal was detected using a Sony CCD camera. In-gel tryptic digest followed by nano-liquid chromatography–tandem mass spectrometry analysis was used to confirm the identity of the band.

Dot blots were used to determine the direct binding of radiolabeled ferrioxamine to crude protein extracts or Fob proteins purified by immunoprecipitation using anti-Fob2p antibodies. Briefly, anti-Fob2p antibodies (1:10 dilution) were coupled to protein G column according to the manufacturer’s recommendation (Thermo Scientific). Protein extracts (100 μl), or bovine serum albumin (BSA) were added to the column at room temperature for 1 h after which the flow through is discarded followed by washing the column 3 times with PBS. ^55^Fe^3+^ labeled ferrioxamine was then added to the anti-Fob2 antibodies-coupled protein G column and incubated for another 1 h at room temperature. Next, the flow through is discarded and the resin was washed three times with PBS before blotting beads containing antibody/antigen complex on PVDF membrane followed by exposure to X-ray film.

### *In vivo* virulence studies

The contribution of *FOB* to *R*. *oryzae* virulence was determined using both a deferoxamine-treated and DKA mouse models. In both models, ICR mice (≥20 g) (Taconic, USA) were used. For the deferoxamine-treated mouse model we adapted the method of Abe et al [64]. Briefly, mice were injected i.p. with three doses of deferoxamine (100 mg/kg/per dose) given on days -1, 0, and +1 relative to infection with *R*. *oryzae*. For the DKA mouse model, mice were rendered diabetic in slight ketoacidosis with a single i.p. injection of 210 mg/kg streptozotocin in 0.2 ml citrate buffer 10 days prior to fungal challenge as we previously described [20,65,66,67]. Glycosuria and ketonuria were confirmed in all mice 7 days after streptozotocin treatment. In both models, mice were infected with 10^5^ spores by intravenous injection in the tail vein. Additionally, in the deferoxamine-treated model, a lower inoculum of 10^3^ was tested. The primary efficacy endpoint was time to moribundity. In some experiments, as a secondary endpoint, fungal burden in the brains and kidneys (primary and secondary target organs) was determined 48 hours after infection by quantitative PCR assay. Briefly, mouse organs were disrupted/homogenized in Lysing Matrix tubes (MP Biomedicals) using FastPrep FP120 Homogenizer (Thermo Electron Corporation), total DNA was then isolated according to the instructional manual of DNeasy Tissue Kit (Qiagen). qPCR was then carried out as previously described [68], using *R*. *oryzae*-specific 18S rRNA primers (S3 Table). Values were expressed as log_10_ spore equivalent/g tissue. Histopathological examination was carried out on sections of the harvested organs after fixing in 10% zinc formalin. The fixed organs were embedded in paraffin, and 5-mm sections were stained with H&E to detect *R*. *oryzae* hyphae [20].

For in vivo expression of *FOB* genes, brains and kidneys were collected from mice 48 hours after wild-type, empty plasmid–transformed, or RNA-i transformed *R*. *oryzae* infection were flash frozen in liquid nitrogen, disrupted/homogenized as above, and then processed for RNA extraction using a Tri Reagent solution (Ambion). Reverse transcription was performed with RETROscript (Ambion) using primers listed in S3 Table. For quantitative RT-PCR, SYBR green assays were performed. Constitutively expressed *ACT1* was used as a control for all reactions. Calculations and statistical analyses were performed using StepOne Real-Time PCR System (Applied Biosystems).

Also, the contribution of the reductase/permease pathway to the pathogenesis of *R*. *oryzae* in the deferoxamine-treated mouse model was determined by comparing the virulence of *R*. *oryzae* with reduced copy number of *FTR1* or with reduced expression of *FTR1* due to RNA-i targeting *FTR1* [24], to their corresponding control strains of *PyrF-*complemented or *R*. *oryzae* transformed with the empty plasmid, respectively. Infection was carried out as above with a targeted inoculum of 1 x 10^3^ spores. The primary efficacy endpoint was time to moribundity.

### Ethics statement

All procedures involving mice were approved by the IACUC of the Los Angeles Biomedical Research Institute at Harbor-UCLA Medical Center (protocol # 11671-08), according to the NIH guidelines for animal housing and care.

### Statistical analysis

Differences in *FOB* expression, growth rates, germination, reductase activity, iron uptake, and tissue fungal burden were compared by the nonparametric Wilcoxon rank-sum test. The nonparametric log-rank test was used to determine differences in survival times. A *P* value <0.05 was considered significant.

## Supporting Information

S1 Fig Predicted cell surface localization of Fob1 (A) and Fob2 (B) by using the MEMSAT program. Fob proteins were predicted to have an extracellular and transmembrane domains as well as cytoplasmic tail.
(TIF)

S2 Fig SDS-PAGE gels of total proteins extracted from wild-type or *R*. *oryzae* dual inhibition mutant. (A) Whole cell extracts from wild-type or *R*. *oryzae fob1/fob2* dual inhibition mutant grown in the presence of absence or ferrioxamine. (B) Cell-free supernatant from cultures of *R*. *oryzae* wild-type or *fob1/fob2* dual inhibition mutants grown in the presence or absence of ferrioxamine. (C) Immunoprecipitated samples of whole cell extracts from wild-type or *R*. *oryzae fob1/fob2* dual inhibition mutant grown in the presence or absence of ferrioxamine using anti-Fob2p antibodies. (D) Proteins from beads coated with anti-rFob2p antibodies and subjected to whole cell extracts from wild-type cells grown in the presence of ferrioxamine. In all gels, rFob2p expressed in *E*. *coli* was used as a control. Bands highlighted with boxes were sequenced and the sequence data is presented in Table 3.
(TIF)

S3 Fig *R*. *oryzae* cell surface localization of Fob proteins when cells are grown in medium containing ferrioxamine. Wild-type cells or cells transformed with empty or RNA-i plasmids were grown in medium containing ferrioxamine as a sole source of iron prior to incubating with sera collected from mice immunized with recombinantly produced Fob2p. Pre immune serum from the same mouse was used as a control. Cells were counterstained with Alexa 488 labeled anti-mouse goat antibody prior to imaging the cells with confocal microscopy. Scale bar = 30 μM.
(TIF)

S4 Fig RNA-i dual construct targeting *FOB1* and *FOB2* did not alter growth of *R*. *oryzae* on iron-rich medium. *R*. *oryzae* 99-880 (wild-type), *R*. *oryzae* transformed with empty plasmid or RNA-i construct were grown at 10 fold dilution (10^5^-10^2^) on YNB medium without uracil. Plates were incubated for 16 h at 37°C before measuring the diameter of the colony. Data (n = 6) are presented as the average colony diameter (mm) + SD of the 10^5^ inoculum.
(TIF)

S5 Fig *R*. *oryzae* iron uptake from ferrioxamine is mediated by reductase activity. (A) The ferrous chelator bipyridyl inhibits growth of *R*. *oryzae* on medium supplemented with ferrioxamine as a sole source of iron (n = 6 per group and per time point). *P <0.006 vs. without bipyridyl, while **P <0.006 vs. without bipyridyl or 0.2 mM bipyridyl. (B) Bipyridyl inhibits ^55^Fe uptake from ferrioxamine (n = 8 per group and per each time point). * P<0.04 vs. without bipyridyl and **P <0.003 vs. without bipyridyl or with 0.2 mM bipyridyl. Error bars represent the standard deviation of the mean from two independent assays.
(TIF)

S1 Table Potential *SIT* genes of *R*. *oryzae* 99-880 identified by % amino acid identity with *S*. *cerevisiae SIT* genes. (DOCX)

S2 Table Strains used in this study. (DOCX)

S3 Table Oligonucleotides used in this study. (DOCX)
