# Supplementary figures and images for: Fob1 and Fob2 Proteins Are Virulence Determinants of Rhizopus oryzae via Facilitating Iron Uptake from Ferrioxamine
Source: PLoS Pathog. 2015 May 14;11(5):e1004842. doi: 10.1371/journal.ppat.1004842 (PMC4431732; doi:10.1371/journal.ppat.1004842)

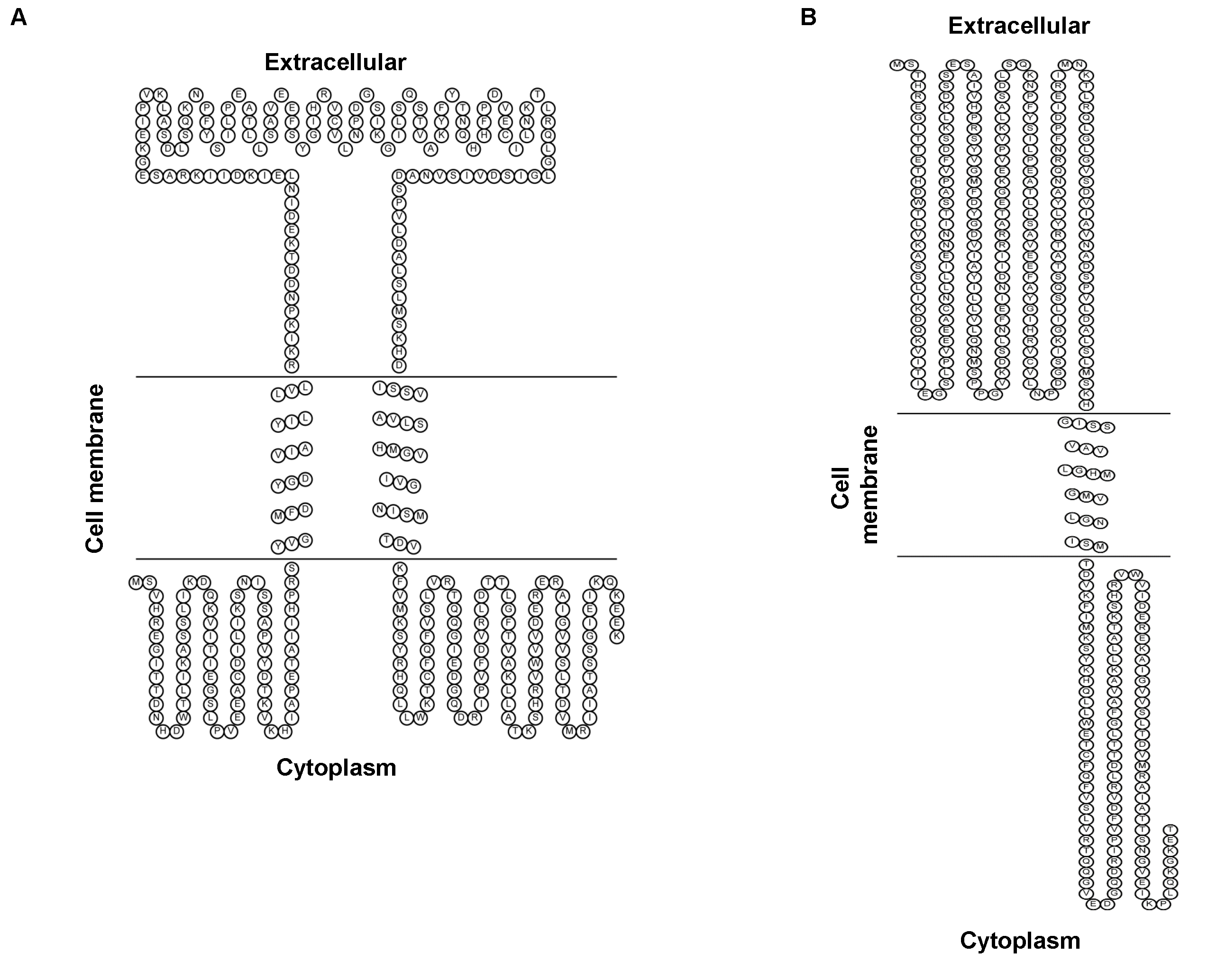

Supplement: S1 Fig — Fob proteins were predicted to have an extracellular and transmembrane domains as well as cytoplasmic tail. (TIF) [file ppat.1004842.s001.tif]

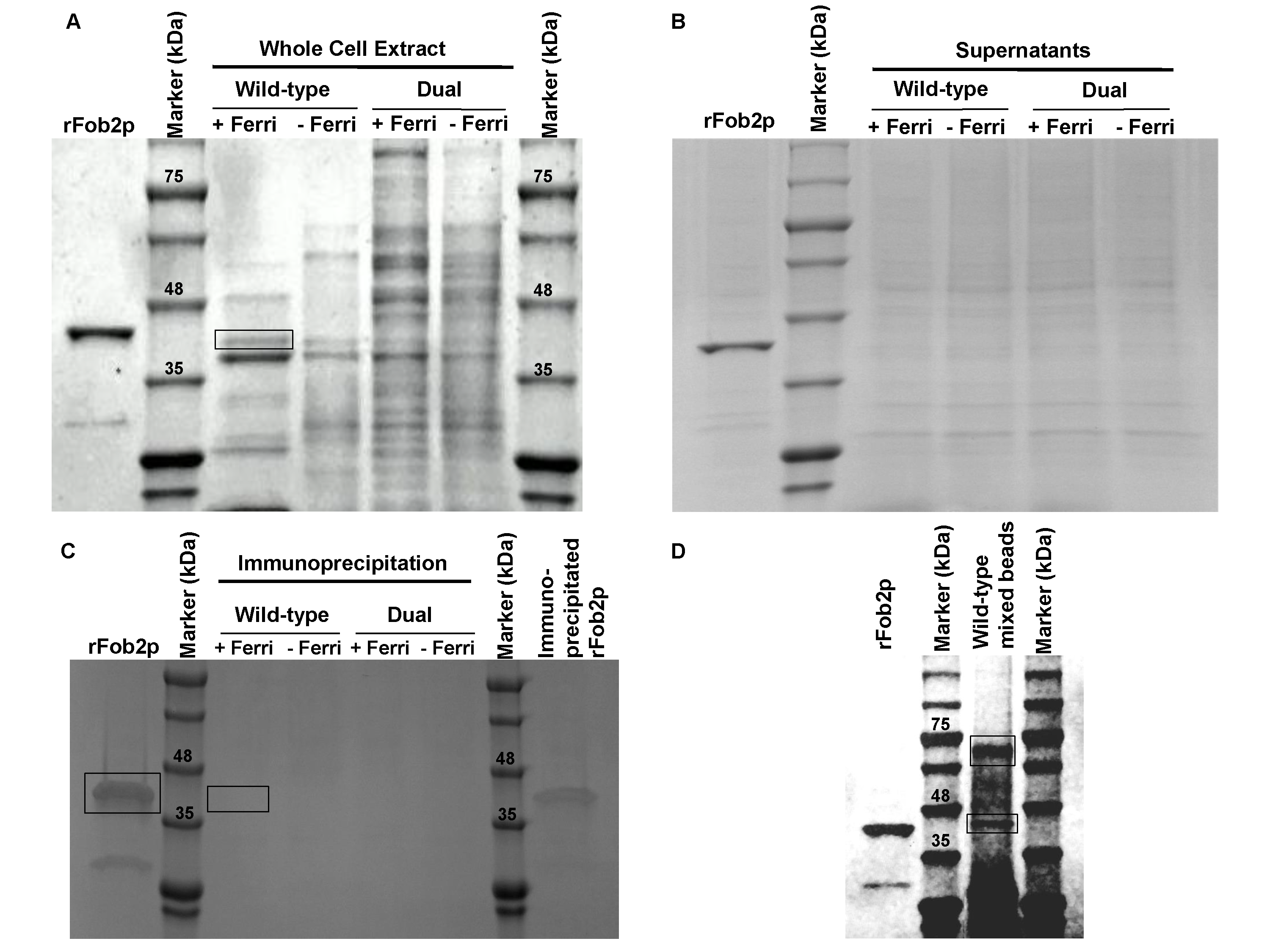

Supplement: S2 Fig — (A) Whole cell extracts from wild-type or R. oryzae fob1/fob2 dual inhibition mutant grown in the presence of absence or ferrioxamine. (B) Cell-free supernatant from cultures of R. oryzae wild-type or fob1/fob2 dual inhibition mutants grown in the presence or absence of ferrioxamine. (C) Immunoprecipitated samples of whole cell extracts from wild-type or R. oryzae fob1/fob2 dual inhibition mutant grown in the presence or absence of ferrioxamine using anti-Fob2p antibodies. (D) Proteins from beads coated with anti-rFob2p antibodies and subjected to whole cell extracts from wild-type cells grown in the presence of ferrioxamine. In all gels, rFob2p expressed in E. coli was used as a control. Bands highlighted with boxes were sequenced and the sequence data is presented in Table 3. (TIF) [file ppat.1004842.s002.tif]

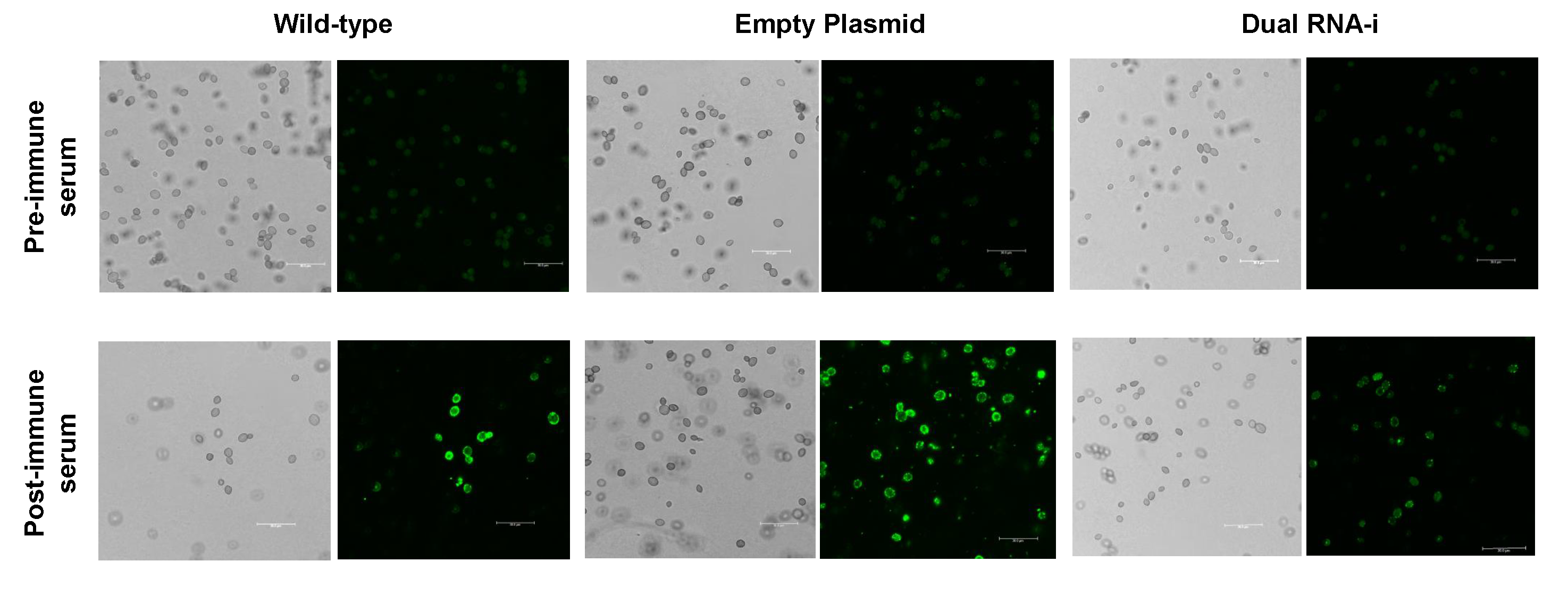

Supplement: S3 Fig — Wild-type cells or cells transformed with empty or RNA-i plasmids were grown in medium containing ferrioxamine as a sole source of iron prior to incubating with sera collected from mice immunized with recombinantly produced Fob2p. Pre immune serum from the same mouse was used as a control. Cells were counterstained with Alexa 488 labeled anti-mouse goat antibody prior to imaging the cells with confocal microscopy. Scale bar = 30 μM. (TIF) [file ppat.1004842.s003.tif]

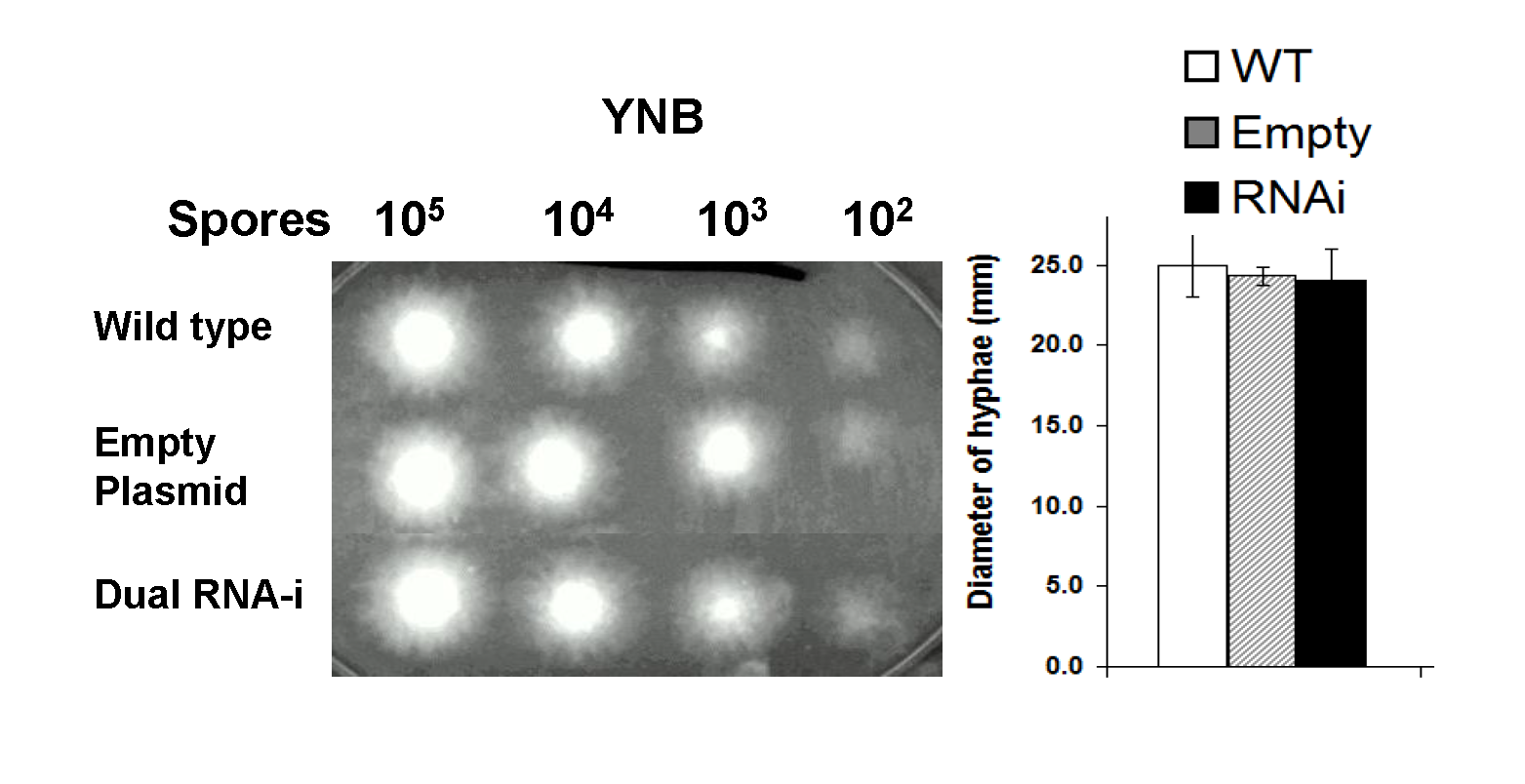

Supplement: S4 Fig — R. oryzae 99-880 (wild-type), R. oryzae transformed with empty plasmid or RNA-i construct were grown at 10 fold dilution (105-102) on YNB medium without uracil. Plates were incubated for 16 h at 37°C before measuring the diameter of the colony. Data (n = 6) are presented as the average colony diameter (mm) + SD of the 105 inoculum. (TIF) [file ppat.1004842.s004.tif]

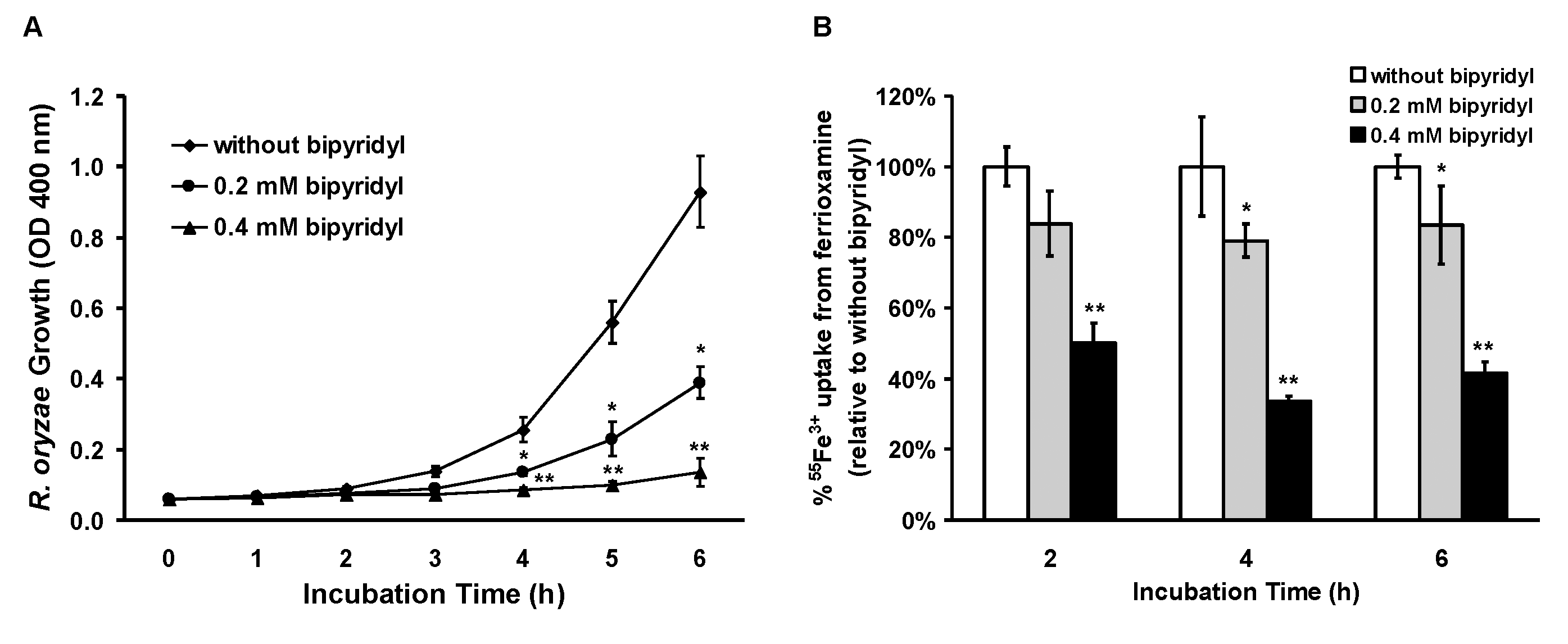

Supplement: S5 Fig — (A) The ferrous chelator bipyridyl inhibits growth of R. oryzae on medium supplemented with ferrioxamine as a sole source of iron (n = 6 per group and per time point). *P <0.006 vs. without bipyridyl, while **P <0.006 vs. without bipyridyl or 0.2 mM bipyridyl. (B) Bipyridyl inhibits 55Fe uptake from ferrioxamine (n = 8 per group and per each time point). * P<0.04 vs. without bipyridyl and **P <0.003 vs. without bipyridyl or with 0.2 mM bipyridyl. Error bars represent the standard deviation of the mean from two independent assays. (TIF) [file ppat.1004842.s005.tif]
