# Supplementary material for: Fob1 and Fob2 Proteins Are Virulence Determinants of Rhizopus oryzae via Facilitating Iron Uptake from Ferrioxamine
Source: PLoS Pathog. 2015 May 14;11(5):e1004842. doi: 10.1371/journal.ppat.1004842 (PMC4431732; doi:10.1371/journal.ppat.1004842)
Supplement: S1 Table — (DOCX) [file ppat.1004842.s006.docx]

**S1 Table.** **Potential *SIT* genes of *R. oryzae* 99-880 identified by % amino acid identity with *S. cerevisiae SIT* genes.**

| ***R. oryzae* ORFs** | ***R. oryzae* given name** | ***S. cerevisiae*** | | | |
| --- | --- | --- | --- | --- | --- |
|  |  | ***ARN1*** | ***ARN2*** | ***ARN3/SIT1*** | ***ARN4*** |
| R03G_00075 | *SIT1* | 21 | 22 | 22 | 23 |
| R03G_02779 | *SIT2* | 18 | 19 | 20 | 17 |
| R03G_02798 | *SIT3* | 18 | 20 | 20 | 20 |
| R03G_05990 | *SIT4* | 23 | 22 | 23 | 22 |
| R03G_09431 | *SIT5* | 17 | 17 | 16 | 15 |
| R03G_11434 | *SIT6* | 18 | 19 | 20 | 18 |
| R03G_12627 | *SIT7* | 20 | 20 | 18 | 21 |
| R03G_16094 | *SIT8* | 10 | 9 | 8 | 10 |
| R03G_16758 | *SIT9* | 22 | 23 | 21 | 20 |
