# Supplementary material for: Fob1 and Fob2 Proteins Are Virulence Determinants of Rhizopus oryzae via Facilitating Iron Uptake from Ferrioxamine
Source: PLoS Pathog. 2015 May 14;11(5):e1004842. doi: 10.1371/journal.ppat.1004842 (PMC4431732; doi:10.1371/journal.ppat.1004842)
Supplement: S2 Table — (DOCX) [file ppat.1004842.s007.docx]

**S2** **Table. Strains used in this study.**

| **Strain** | **Genotype** | **Description and source** |
| --- | --- | --- |
| *R. oryzae* 99–880 | Wild-type | Clinical isolate [20] |
| *R. oryzae* M16 | *pyrF205* | Uracil deficient [20,52] |
| *R. oryzae PyrF-*complemented | *pyrF205::PyrF* | M16 complemented with a wild-type copy of *PyrF* at its original locus [24] |
| *R. oryzae FTR1* reduced copy number (KO) | *pyrF205, ftr1::PyrF* | *ftr1* knock out strain with reduced copy number [24] |
| *R. oryzae* Empty | M16 (pRNAi-pdc intron) | M16 transformed with empty plasmid [24] |
| *R. oryzae* FTR1Inh | M16 (p*FTRi-pdc* intron) | *FTR1* inhibited by RNAi [24] |
| *R. oryzae FOB1* | M16 (p*FOB1-pdc* intron) | *FOB1* inhibited by RNAi, this work |
| *R. oryzae FOB2* | M16 (p*FOB1-pdc* intron) | *FOB2* inhibited by RNAi, this work |
| *R. oryzae* dual | M16 (p*FOB1/FOB2-pdc* intron) | Both *FOB1* and *FOB2* inhibited by RNAi, this work |
| *E. coli* XL-10 gold | Tet^r^ Δ(*mcrA*)*183* Δ(*mcrCB-hsdSMR-mrr*)*173 endA1 supE44 thi-1 recA1 gyrA96 relA1 lac* Hte [F´ *proAB lacI*^q^*Z*Δ*M15* Tn*10* (Tet^r^) Amy Cam^r^] | Host for transformation and protein expression, Agilent Technologies. |
