# Supplementary material for: Fob1 and Fob2 Proteins Are Virulence Determinants of Rhizopus oryzae via Facilitating Iron Uptake from Ferrioxamine
Source: PLoS Pathog. 2015 May 14;11(5):e1004842. doi: 10.1371/journal.ppat.1004842 (PMC4431732; doi:10.1371/journal.ppat.1004842)
Supplement: S3 Table — (DOCX) [file ppat.1004842.s008.docx]

**S3 Table. Oligonucleotides used in this study.**

| **Primers** | **Sequences (5’-3’)** | **Description** |
| --- | --- | --- |
| F-*ACT1* | AAGACGAAGTTGCTGCTCTTG | actin, F for qPCR |
| R- *ACT1* | GACGCACCTCGTGCTGTCTT | actin, R for qPCR |
| Def05087-1 | ACGTGCATGCATATGTCTGTTCACAGAGAAG | For cloning FOB1 RNA-i fragment into RNA-i plasmid pRNAi-pdc-intron (pair with Def05087-2), forward fragment. |
| Def05087-2 | TGGCGCGCCTTCTACAGCAGATAATAAAG | R primer. |
| Def05087-3 | TGCTAGCATGTCTGTTCACAGAGAAGGGA | For cloning FOB1 RNA-i fragment into RNA-i plasmid pRNAi-pdc-intron (pair with Def05087-4), inverted fragment |
| Def05087-4 | TCCGCGGTTCTACAGCAGATAATAAAGTAG | R primer |
| Def11000-1 | ACGTGCATGCATATGTCTACTCATAGAGAAGGAA | For cloning FOB2 RNA-i fragment into RNA-i plasmid pRNAi-pdc-intron (pair with Def11000-2), forward fragment |
| Def11000-2 | TAGTGGCGCGCCAAATTCCTCTACAGCAGA | R primer |
| Def11000-3 | ACGTGCTAGCATGTCTACTCATAGAGAAGGAA | For cloning FOB2 RNA-i fragment into RNA-i plasmid pRNAi-pdc-intron (pair with Def11000-4), inverted fragment |
| Def11000-4 | TACTCCGCGGAAATTCCTCTACAGCAGAC | R primer |
| 18S-F | GCGGATCGCATGGCC | 18S rRNA, F for qPCR |
| 18S-R | CCATGATAGGGCAGAAAATCG | 18S rRNA, R for qPCR |
| Def05087RT-fwd | TAGCAAAGTACTTTTATACAAACCAACATC | FOB1, F for qPCR |
| Def05087RT-rev | TACCAAGACCTAATTGGCGCAAA | FOB1, R for qPCR |
| Def11000RT-fwd | AGATATCTTTATGCAAATCAGCGCA | FOB2, F for qPCR |
| Def11000RT-rev | CTCCAAGACCTAACTGACGTAGT | FOB2, R for qPCR |
| FOB2a | GTACGGATCCTGTCTACTCATAGAGAAGGAATTAC | For cloning FOB2 in E. coli, BamHI site underlined |
| FOB2b | TCGACTGCAGTTATGTTTCTTTCCCTTTTTGCAAAG | For cloning FOB2 in E. coli, PstI site underlined |
| SIT1a | ATACCTTAGTCGGTGGCCAAGT | SIT1, F for qPCR |
| SIT1b | CCATCTCCAAGCATTAGCATTC | SIT1, R for qPCR |
| SIT4a | TGTATCACTGCAACAGCCATTC | SIT4, F for qPCR |
| SIT4b | CAGTGGCAGCAAAATCATACAA | SIT4, R for qPCR |
| SIT6a | AAACCAGAGCTGACGATGAGAG | SIT6, F for qPCR |
| SIT6b | AAAGCATCAAAGGTCAGCATGT | SIT6, R for qPCR |
| SIT9a | AGGTGTCCAGGCATCTAGTCAA | SIT9, F for qPCR |
| SIT9b | GGAAGAACTATAAGGCCCGAGA | SIT9, R for qPCR |

F, forward; R, reverse.
